# Role of hormones and neurosteroids in epileptogenesis

**DOI:** 10.3389/fncel.2013.00115

**Published:** 2013-07-31

**Authors:** Doodipala Samba Reddy

**Affiliations:** Department of Neuroscience and Experimental Therapeutics, College of Medicine, Texas A&M University Health Science CenterBryan, TX, USA

**Keywords:** epilepsy, epileptogenesis, neurosteroid, estrogen, progesterone, kindling

## Abstract

This article describes the emerging evidence of hormonal influence on epileptogenesis, which is a process whereby a brain becomes progressively epileptic due to an initial precipitating event of diverse origin such as brain injury, stroke, infection, or prolonged seizures. The molecular mechanisms underlying the development of epilepsy are poorly understood. Neuroinflammation and neurodegeneration appear to trigger epileptogenesis. There is an intense search for drugs that truly prevent the development of epilepsy in people at risk. Hormones play an important role in children and adults with epilepsy. Corticosteroids, progesterone, estrogens, and neurosteroids have been shown to affect seizure activity in animal models and in clinical studies. However, the impact of hormones on epileptogenesis has not been investigated widely. There is emerging new evidence that progesterone, neurosteroids, and endogenous hormones may play a role in regulating the epileptogenesis. Corticosterone has excitatory effects and triggers epileptogenesis in animal models. Progesterone has disease-modifying activity in epileptogenic models. The antiepileptogenic effect of progesterone has been attributed to its conversion to neurosteroids, which binds to GABA-A receptors and enhances phasic and tonic inhibition in the brain. Neurosteroids are robust anticonvulsants. There is pilot evidence that neurosteroids may have antiepileptogenic properties. Future studies may generate new insight on the disease-modifying potential of hormonal agents and neurosteroids in epileptogenesis.

## INTRODUCTION

Epilepsy, one of the most common serious neurological disorders, is characterized by the unpredictable occurrence of seizures. A seizure is an abnormal electrical discharge in the brain that causes an alteration in consciousness, sensations, and behaviors. The symptoms that occur depend on the parts of the brain affected during the seizure. Common signs of seizures include staring, unusual feelings, twitching, unconsciousness, and jerking in the arms or legs. Epilepsy affects an estimated 3 million Americans and about 65 million people worldwide in a variety of ways (Jacobs et al., 2009; Hesdorffer et al., 2013). About 150,000 new cases of epilepsy are diagnosed in the United States annually (Hesdorffer et al., 2013). Children and adults are the fastest-growing segments of the population with new cases of epilepsy.

Epilepsy is a collective designation for a group of brain disorders consisting of a complex spectrum of different seizure types and syndromes. Epileptic seizures are classified into partial (simple and complex partial seizures) and generalized seizures (absence, tonic–clonic, myoclonic, and atonic seizures). Accurate diagnosis of seizure type and epileptic syndrome is critical for determining appropriate drug therapy and prognosis. The International League Against Epilepsy (ILAE) provided a definition of “seizure” and “epilepsy” (Fisher et al., 2005). A seizure is defined as “a transient occurrence of signs and/or symptoms due to abnormal synchronous neuronal activity in the brain.” Epilepsy is defined as “a disorder of the brain characterized by an enduring predisposition to generate epileptic seizures.” A single seizure, therefore, does not constitute epilepsy. The diagnosis of epilepsy requires the occurrence of recurrent (two or more) epileptic seizures separated by at least 24 h, unprovoked by any immediate identified cause. Antiepileptic drugs (AEDs) are the mainstay for controlling seizures (**Table 1**). Current drug therapy is symptomatic in that available drugs inhibit seizures, but neither effective prophylaxis nor cure are available. The goal of the therapy is to eliminate seizures without interfering with normal function (Glauser et al., 2006, 2013). Despite many advances in epilepsy research, presently an estimated 30% of people with epilepsy have “intractable seizures” that do not respond to even the best available medication. There is renewed focus on the pathophysiology of epileptogenesis, the process whereby a brain becomes progressively epileptic due to an initial precipitating event.

**Table 1:** List of current antiepileptic drugs.

Standard (first generation)	Newer (second generation)
Carbamazepine (Tegretol)	Acetazolamide (Diamox)
Clonazepam (Klonopin)	Clobazam (Onfi)
Chlorazepate (Tranxene)	Ezogabine (Potiga)
Diazepam (Valium)	Felbamate (Felbatol)
Divalproex sodium (Depakote)	Fosphenytoin (Cerebyx)
Ethosuximide (Zarontin)	Lacosamide (Vimpat)
Ethotoin (Peganone)	Lamotrigine (Lamictal)
Lorazepam (Ativan)	Levetiracetam (Keppra)
Mephobarbital (Mebaral)	Oxcarbazepine (Trileptal)
Methsuximide (Celontin)	Parampanel (Fycompa)
Nitrazepam (Mogadon)	Pregabalin (Lyrica)
Phenobarbital (Gardinal)	Progabide (Gabrene)
Phenytoin (Dilantin)	Rufinamide (Banzel)
Primidone (Mysoline)	Tiagabine (Gabitril)
Valproic acid (Depakene)	Topiramate (Topamax)
	Vigabatrin (Sabril)
	Zonisamide (Zonegran)

This article describes the emerging evidence of hormonal influence on epileptogenesis and the potential mechanisms underlying their actions on neuronal excitability and seizure activity. It also describes recent studies on neurosteroid agents that prevent or delay the development of epilepsy. The main focus of the review is on steroid hormones and neurosteroids with seizure-modulating activity. Neuropeptides and other hormones such as oxytocin, neuropeptide-Y, and galanin, which may affect neuronal excitability, are not discussed here because such description is beyond the scope of this article. The seizure-modulating effects of neuropeptides are discussed elsewhere (Robertson et al., 2011).

## OVERVIEW OF EPILEPTOGENESIS AND INTERVENTION STRATEGIES

Epilepsy is a chronic condition with many possible causes. Epilepsy may develop because of an abnormality in neural connectivity, an imbalance in inhibitory and excitatory neurotransmitters, or some combination of these factors. Primary epilepsy (50%) is idiopathic (“unknown cause”). In secondary epilepsy (50%), seizures may result from a variety of conditions including trauma, anoxia, metabolic imbalances, tumors, encephalitis, drug withdrawal, and neurotoxicity (Engel et al., 2007). The molecular mechanisms underlying the development of acquired epilepsy are not very well understood. The term “epileptogenesis” is used to describe the complex plastic changes in the brain that, following a precipitating event, convert a normal brain into a brain debilitated by recurrent seizures (Pitkänen et al., 2009; Pitkänen and Lukasiuk, 2011). Although specific types of epilepsy may have unique pathophysiological mechanisms, a broad hypothesis in this field is that convergent neuronal mechanisms are common in different forms of acquired epilepsy.

**FIGURE 1:**
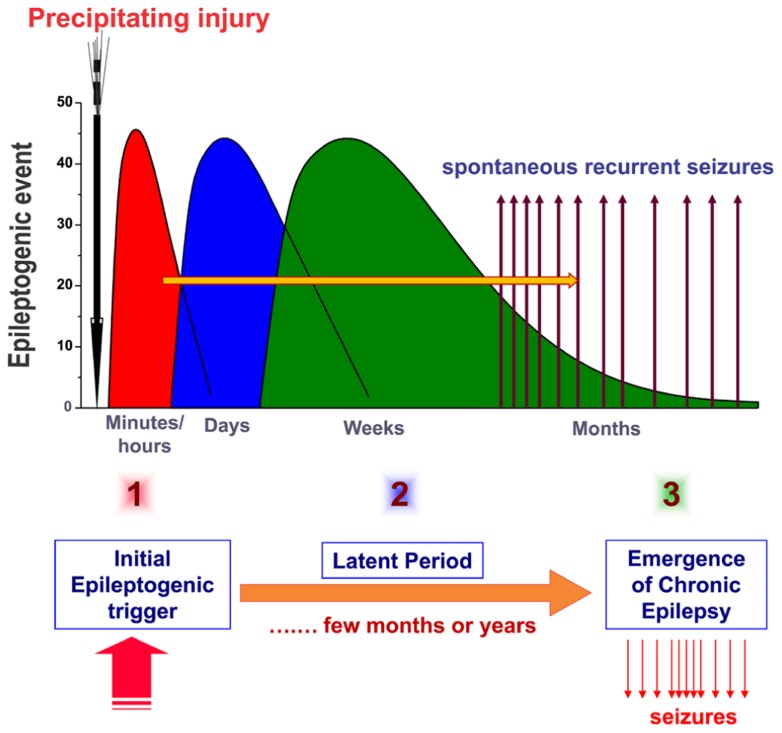
**Pathophysiology of epileptogenesis.** Epileptogenesis is the process whereby a normal brain becomes progressively epileptic because of precipitating injury or risk factors such as TBI, stroke, brain infections, or prolonged seizures. Epilepsy development can be described in three stages: (1) the initial injury (epileptogenic event); (2) the latent period (silent period with no seizure activity); and (3) chronic period with spontaneous recurrent seizures. Although the precise mechanisms underlying spatial and temporal events remain unclear, epileptogenesis may involve an interaction of acute and delayed anatomic, molecular, and physiological events that are both complex and multifaceted. The initial precipitating factor activates diverse signaling events, such as inflammation, oxidation, apoptosis, neurogenesis, and synaptic plasticity, which eventually lead to structural and functional changes in neurons. These changes are eventually manifested as abnormal hyperexcitability and spontaneous seizures.

The current hypothesis about the pathogenesis of epilepsy (epileptogenesis) involves three stages: (1) the initial precipitating event; (2) the latent period (no seizures); and (3) the chronic period with spontaneous seizures (**Figure 1**). Acquired epilepsy typically develops due to an initial precipitating event such as traumatic brain injury (TBI), stroke, brain infections, or prolonged seizures. The other possible precipitating triggers for epileptogenesis include febrile seizures, metabolic dysfunction, alcohol withdrawal, and status epilepticus, an emergency condition characterized by continuous seizures or repeated seizures without regaining consciousness for 30 min or more (McClelland et al., 2011). Exposure to organophosphorous pesticides and chemical warfare nerve agents, such as soman, can cause epilepsy as a result of cholinergic neurotoxicity and status epilepticus (de Araujo Furtado et al., 2012).

The development of epileptogenesis is thought to be a step-function of time after the brain injury, with a latent period present between the brain injury and the first unprovoked seizure. There are some alternative hypotheses to this notion that view epileptogenesis as a continuous process that extends past the first spontaneous seizure (Dudek and Staley, 2011). Temporal lobe epilepsy (TLE) is one of the common forms of chronic epilepsies (Wieser and ILAE Commission on Neurosurgery of Epilepsy, 2004). TLE is characterized by the progressive expansion of spontaneous seizures originating from the limbic system regions, especially the hippocampus, most often due to neuronal injury. The hippocampal sclerosis, which is characterized by aberrant mossy fiber sprouting and widespread neuronal loss in the dentate hilus and CA1 and CA3 subfields, is the hallmark of epilepsy pathology (Sutula et al., 1989; Buckmaster et al., 2002; Nadler, 2003; Morimoto et al., 2004). However, there is an ongoing debate about whether the hippocampal sclerosis is the basis or the outcome of recurring seizures. There is emerging evidence from refractory models that epilepsy involves “progressive epileptogenesis” much beyond the latent period and the onset of the first seizure (Williams et al., 2009). Several mechanisms have been described including loss of interneurons (van Vliet et al., 2004; Sloviter and Bumanglag, 2013) and neuroinflammation (Vezzani et al., 2011). There is great variation in the onset of spontaneous seizures following a precipitating factor (Rao et al., 2006; Norwood et al., 2010). Thus, the critical window for effective “antiepileptogenic” interventions remain poorly defined for curing epilepsy in people at risk.

Studies in animal models have provided improved understanding of neurophysiological basis of epileptic seizures (O’Dell et al., 2012; Simonato et al., 2012). Spontaneous seizures arise from hyperexcitable and hypersynchronous neuronal networks and involve both cortical and several key subcortical structures. The general cellular pathway underlying occurrence of epileptic seizures is apparent in three phases: (i) focal epileptogenicity (initiation); (ii) synchronization of the surrounding neurons (sync); and (iii) propagation of the seizure discharge to other areas of the brain (spread). Experimental field and intracellular recordings in isolated brain sections provide a detailed description of neurophysiological abnormalities underlying epileptic regions. Paroxysmal depolarization shift (PDS) is identified as a hallmark of epileptic neurons. It is characteristic of neurons in epileptic cortical zones and consists of an abnormally prolonged depolarization with repetitive spiking reflected as interictal discharges in the electroencephalogram (EEG). High-frequency oscillations, termed ripples (80–200 Hz) and fast ripples (250–600 Hz), are recorded in the EEG of epileptic patients and in animal epilepsy models (Köhling and Staley, 2011; Lévesque et al., 2011). Fast ripples are thought to reflect pathological activity and seizure onset zones.

Gowers (1881) proposed that *seizures beget seizures*. The kindling model has provided a conceptual framework for this idea and for developing new molecular targets for preventing epilepsy (Goddard et al., 1969; McNamara et al., 1992). Post-status epilepticus paradigms are widely used for modeling the epileptogenesis in which a single episode of prolonged seizures (by pilocarpine, kainate, or electrical stimulation) triggers progressive development of seizure activity (Buckmaster and Dudek, 1997; Hellier et al., 1999; Glien et al., 2001; Rao et al., 2006; Löscher, 2012). These chronic models share many features of human limbic epilepsy (Löscher, 2002; Stables et al., 2002). Pilocarpine, kainate, or perforant path stimulation induce acute SE and neuronal injury and follow a pattern of latent period similar to that observed in limbic epilepsy. Like TLE, mossy fiber sprouting, neurodegeneration, and ectopic granule cell proliferation are evident after the latent period (Löscher, 2002; Rao et al., 2006).

Despite decades of research, currently there is no single Food and Drug Administration (FDA)-approved drug that truly prevents the development of epilepsy in people at risk. A variety of intervention approaches have been tested in animal models of epileptogenesis (Acharya et al., 2008; Pitkänen and Lukasiuk, 2011). A number of clinical trials show a lack of antiepileptogenic efficacy of AEDs, including phenytoin and carbamazepine, in patients at high risk for developing epilepsy (Temkin, 2001; Mani et al., 2011). There is a desperate need for drugs that truly prevent the development of epilepsy (“antiepileptogenic agents”) or alter its natural course to delay the appearance or severity of epileptic seizures (“disease-modifying agents”).

In 2000, National Institute of Neurological Disorders and Stroke (NINDS) and epilepsy research and advocacy groups organized the first “Curing Epilepsy” conference, which marked a turning point for shifting and expanding the focus of epilepsy research toward cures for epilepsy and the prevention of epilepsy in those at risk (Jacobs et al., 2001). During the past decade, there has been increasing research emphasis on the prevention of epileptogenesis and translation of lead discoveries in this field into therapies for curing epilepsy (Jacobs et al., 2009; Simonato et al., 2012). The Institute of Medicine (IOM) released a consensus report in 2012 on public health dimensions of the epilepsies focusing on promoting health and understanding epilepsy (Austin et al., 2012; Hesdorffer et al., 2013). The IOM report, *Epilepsy Across the Spectrum: Promoting Health and Understanding*, provided 13 recommendations for future work in the field of epilepsy. The report contains research priorities which include one key recommendation on prevention of epilepsy.

## ROLE OF STEROID HORMONES IN EPILEPTOGENESIS

Steroid hormones play a key role in the neuroendocrine control of neuronal excitability and seizure susceptibility (**Table 2**; Herzog, 2002; Reddy, 2003a, 2010; Verrotti et al., 2007). Steroid hormones are synthesized and secreted from ovarian, gonadal, and adrenal sources. In men, the main circulating steroids are androgenic steroids (testosterone and dihydrotestosterone) and adrenal corticosteroids (cortisol and aldosterone). Deoxycorticosterone (DOC) is also released from adrenal cortex in response to stress. In women, the primary reproductive steroid hormones are estrogens and progesterone, which are released during the menstrual cycle. The early follicular phase is associated with low levels of estrogens and progesterone. The synthesis and secretion of estrogens and progesterone from the ovaries are controlled primarily by hypothalamic gonadotropin releasing hormone (GnRH) and the pituitary gonadotropins, follicle stimulating hormone (FSH) and luteinizing hormone (LH). As ovulation approaches, the level of estrogen rises and triggers a large surge of LH leading to ovulation. Following ovulation, the ruptured follicle luteinizes and forms a corpus luteum that secretes progesterone and estrogen. Estradiol is secreted in the second half of the follicular phase and increases to a peak at midcycle, while progesterone is elevated during the luteal phase and declines before menstruation begins.

**Table 2:** List of steroid hormones and neurosteroids that affect seizure susceptibility.

Anticonvulsant steroids	Proconvulsant steroids
Progesterone	Estradiol
Allopregnanolone	Pregnenolone sulfate
Pregnanolone	DHEA sulfate
Dihydroprogesterone	Cortisol
Androstanediol	11-Deoxycortisol
Etiocholanone	
Dihydrotestosterone	
Deoxycorticosterone	
Dihydrodeoxycorticosterone	
Allotetrahydrodeoxycorticosterone	

The cyclical changes of estrogens and progesterone are now widely believed to be important in the pathogenesis of catamenial epilepsy, a menstrual cycle-related seizure disorder in women with epilepsy (Reddy, 2009a, 2013). Catamenial epilepsy is a multifaceted neuroendocrine condition in which seizures are clustered around specific points in the menstrual cycle, most often around perimenstrual or periovulatory period. Generally, estrogens are found to be excitatory or proconvulsant, while progesterone has powerful antiseizure effect and reduces seizures, and thus they play a central role in the pathophysiology of epilepsy in women (Reddy, 2009a, 2013). Progesterone is an intermediate precursor for the synthesis of neurosteroids, which are increased in parallel during the ovarian cycle. There is emerging evidence that endogenous neurosteroids influence seizure susceptibility and epileptogenesis (Reddy, 2011; Reddy and Rogawski, 2012).

### PROGESTERONE

**FIGURE 2:**
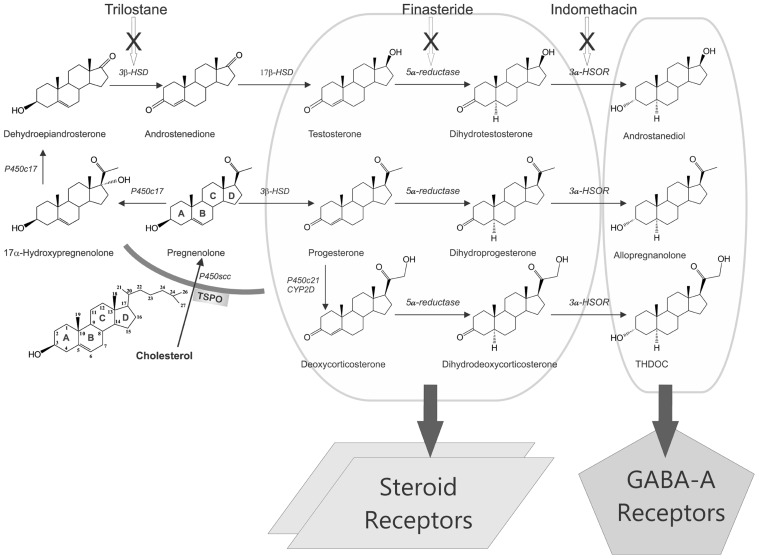
**Biosynthesis and targets of steroid hormones and neurosteroids in the brain.** Enzymatic pathways for the production of three prototype neurosteroids allopregnanolone, THDOC, and androstanediol are illustrated from cholesterol. Steroid hormones progesterone, deoxycorticosterone, and testosterone undergo two sequential A-ring reduction steps catalyzed by 5α-reductase and 3α-HSOR to form the 5α,3α-reduced neurosteroids. The conversion of intermediate precursor steroids into neurosteroids occurs in the hippocampus and several other regions within the brain, where they can affect neuronal function. As evident from the pathways, many modifications are made by the same enzymes, which can be blocked by specific inhibitors (trilostane, finasteride, and indomethacin). There are two mechanisms by which steroid hormones affects neuronal function: (i) binding to steroid receptors (PRs, ARs, or MRs; left panel) and (ii) conversion to GABA-A receptor-modulating neurosteroids (right panel). Progesterone, deoxycorticosterone, or testosterone binding to their cognate steroid receptors could lead to activation of gene expression in the brain. Neurosteroids rapidly modulate neuronal excitability by direct interaction with inhibitory GABA-A receptors in the brain.

Progesterone is an endogenous anticonvulsant hormone with substantial impact on seizure susceptibility. The potential molecular pathways for the progesterone modulation of seizure activity are illustrated in **Figure 2**. Progesterone is an appealing hormone for prophylactic interventions on epilepsy development, due to its multifunctional modulatory actions in the brain. Progesterone has long been known to have antiseizure activity in animal models (Selye, 1942; Craig, 1966; Kokate et al., 1999a; Frye and Scalise, 2000; Reddy et al., 2004), and in clinical studies (Bäckström et al., 1984; Herzog, 1995, 1999). Women with epilepsy are prone to seizures in response to decreased levels of progesterone during perimenstrual periods (Herzog et al., 1997; Reddy, 2009a). Indeed, the incidence of epilepsy is generally lower in women than in men (Hauser et al., 1993; Christensen et al., 2005; McHugh and Delanty, 2008). This gender difference could be caused by ovarian hormones such as progesterone. Although progesterone is known to inhibit stimulation-evoked seizures in kindling models (Holmes and Weber, 1984; Mohammad et al., 1998; Lonsdale and Burnham, 2003), it has not been investigated widely for potential disease-modifying effect in epileptogenic models. In the kindling model, progesterone has been shown to impair or retard epileptogenesis (Holmes and Weber, 1984; Edwards et al., 2001; Reddy et al., 2010).

Recently progesterone has been evaluated in an National Institutes of Health (NIH)-sponsored, multicenter clinical trial as a treatment for epilepsy in women (Herzog et al., 2012). This randomized, double-blind, placebo-controlled, phase III, multicenter, clinical trial compared the efficacy and safety of adjunctive cyclic natural progesterone therapy versus placebo treatment of intractable seizures in 294 subjects randomized 2:1 to progesterone or placebo, stratified by catamenial and non-catamenial status. The results indicate lack of significant difference in proportions of responders between progesterone and placebo groups. However, a more restricted analysis on a subset of the data found a significantly higher responder rate in women with perimenstrual seizure exacerbation. These findings suggest that progesterone may provide a clinically important benefit for a subset of women with perimenstrual catamenial epilepsy. The dramatic response to progesterone, which is a neurosteroid precursor, in women with perimenstrual catamenial epilepsy is attributable to the unique neurosteroid sensitivity of perimenstrual catamenial seizures (Reddy, 2009a).

Previous studies have shown that progesterone supports the normal development of neurons, and that it reduces the extent of brain damage after TBI (Roof et al., 1994; Stein, 2013). It has been observed in animal models that females have reduced susceptibility to TBI and this protective effect has been hypothesized to be caused by increased circulating levels of progesterone in females (Roof and Hall, 2000; Meffre et al., 2007). A number of additional studies have confirmed that progesterone has neuroprotective effects (Gibson et al., 2008; Singh and Su, 2013). Promising results have also been reported in human clinical trials. Recently, two clinical studies have evaluated progesterone as a treatment for moderate to severe TBI (Wright et al., 2007; Xiao et al., 2008). These studies demonstrated the efficacy of progesterone as a neuroprotective agent in TBI. Progesterone is highly efficacious in reducing disability and death in TBI. Progesterone has neuroprotective properties in acute models of ischemic injury, stroke, and astroglial dysfunction (Koenig et al., 1995; Jiang et al., 1996; He et al., 2004), suggesting its beneficial effects in brain injury.

Progesterone targets multiple molecular and cellular mechanisms relevant to epileptogenesis, and may therefore be a natural disease-modifying agent. Progesterone’s cellular actions are mediated by the progesterone receptors (PRs), which are expressed in the hypothalamus, neocortex, hippocampus, and limbic areas (Brinton et al., 2008). Progesterone is an intermediate precursor for the synthesis of neurosteroids (see Role of Neurosteroids in Epileptogenesis). Progesterone’s antiseizure activity is mediated mainly by its conversion to allopregnanolone, a neurosteroid and positive modulator of GABA-A receptors with broad-spectrum antiseizure properties (Belelli et al., 1989; Kokate et al., 1999a; Frye et al., 2002; Kaminski et al., 2004; Reddy et al., 2004). Moreover, neurosteroids can modulate PRs via intracellular metabolism to analogs that bind to PRs (Rupprecht et al., 1993). Progesterone may modulate signaling cascades of inflammation, apoptosis, neurogenesis, and synaptic plasticity (Patel, 2004; Vezzani, 2005; Stein and Sayeed, 2010), and therefore, progesterone may directly exert disease-modifying effects on epileptogenesis.

Recently, progesterone has been tested in rodent models of hippocampus epileptogenesis (Reddy et al., 2010; Reddy and Mohan, 2011; Reddy and Ramanathan, 2012). At low, non-sedative doses, progesterone treatment for 2 weeks significantly suppressed the rate of development of kindled seizure activity evoked by daily hippocampus stimulation in mice, indicating a disease-modifying effect of progesterone on limbic epileptogenesis. There was a significant increase in the rate of “rebound or withdrawal” kindling during drug-free stimulation sessions following abrupt discontinuation of progesterone treatment. A washout period after termination of progesterone treatment prevented such acceleration in kindling. The molecular mechanisms underlying progesterone’s attenuating effect on kindling development remains unknown. There are several potential mechanisms by which progesterone could inhibit epileptogenesis, including activation of PRs, synthesis of neurosteroids, modulation of oxidative cascades, and promoting neuroprotection. The effect of progesterone on the early kindling progression is reduced in mice lacking PRs, which provide evidence that PRs may be partly involved in progesterone’s disease-modifying effects (Reddy et al., 2010). These findings are consistent with the role of PRs in progesterone inhibition of epileptiform activity in the hippocampus (Edwards et al., 2000). Despite the early attenuation, late stage kindling progressed normally in the PRKO mice, suggesting that PRs are not involved in the later part of the kindling progression. However, the extent to which PRs mediate the progesterone’s disease-modifying effect remains unclear. Collectively, the disease-modifying effect of progesterone may occur through a complex mechanism partly involving PR-dependent and PR-independent pathways.

Progesterone is rapidly metabolized into neurosteroids pregnanolone and allopregnanolone, which could mediate progesterone’s attenuating effects on kindling epileptogenesis. This possibility is supported by emerging evidence that neurosteroids can retard the development of spontaneous seizures in post-SE models of epileptogenesis (Biagini et al., 2006, 2009). It is suggested that 5α-reductase converts progesterone to allopregnanolone and related neurosteroids that retard epileptogenesis. To further test this hypothesis, we utilized the mouse hippocampus kindling model of epileptogenesis and investigated the effect of finasteride, a 5α-reductase and neurosteroid synthesis inhibitor (Reddy and Ramanathan, 2012). In a kindling model in adult mice, pre-treatment with finasteride significantly blocked progesterone’s inhibition of epileptogenesis (Reddy and Mohan, 2011), and led to complete inhibition of the progesterone-induced retardation of limbic epileptogenesis in mice (Reddy and Ramanathan, 2012). Therefore, neurosteroids such as allopregnanolone may mediate the disease-modifying effect of progesterone in the kindling model (see Role of Neurosteroids in Epileptogenesis).

In addition, progesterone’s modulation of inflammation is suggested as an appealing mechanism because pro-inflammatory molecules and oxidative signaling has been found to be activated in animal models of epilepsy (Patel, 2004; Vezzani, 2005). Progesterone has pleiotropic effects on inflammation and cell growth/survival (He et al., 2004; Stein and Sayeed, 2010) that may contribute to its attenuating effects on epileptogenesis. Progesterone inhibits secreted phospholipase A2 enzyme, a very high level target in the inflammatory cascade that has been shown to induce neurodegeneration through glutamate release (DeCoster et al., 2002; Yagami et al., 2002). Progesterone has been shown in numerous preclinical models to be neuroprotective after injury (Roof et al., 1994; Koenig et al., 1995; Jiang et al., 1996; Cutler et al., 2007). Recently, two clinical studies have evaluated progesterone as a treatment for moderate to severe TBI (Wright et al., 2007; Xiao et al., 2008). The progesterone was administered over a period of 3 or 5 days beginning within 8 or 11 h of the injury. In both studies, the groups receiving progesterone had significantly fewer deaths than those receiving placebo. In addition, there was evidence of improved functional outcomes in the progesterone-treated groups, suggesting that progesterone is highly efficacious in reducing morbidity and mortality in TBI, which is a leading cause of epilepsy in adults and military persons.

### TESTOSTERONE

Testosterone has marked impact on seizure susceptibility. The potential biosynthetic pathways of testosterone metabolism are illustrated in **Figure 2**. Testosterone is known to produce both proconvulsant and anticonvulsant effects depending on the animal model and the seizure type (Reddy, 2008). Both animal and clinical studies show that testosterone enhances seizure activity by metabolism to estrogens (Isojarvi et al., 1988; Thomas and Yang, 1991; Herzog et al., 1998; Edwards et al., 1999; El-Khayat et al., 2003). Epidemiological data indicate that the occurrence of focal and tonic–clonic epileptic seizures is ~50% higher in intact than in castrated dogs (VMDB Report, 2003). On the contrary, testosterone and related androgens have protective effects against seizures induced by pentylenetetrazol and kainic acid (Schwartz-Giblin et al., 1989; Frye and Reed, 1998; Frye et al., 2001a,b; Reddy, 2004b). Moreover, studies in orchidectomized or castrated animals have shown that decreased testosterone is associated with higher incidence of seizures and replacement with testosterone attenuates seizures (Grigorian and Khudaverkian, 1970; Thomas and McLean, 1991; Pericić et al., 1996; Pesce et al., 2000). It is demonstrated that testosterone modulation of seizure susceptibility occurs through its conversion to neurosteroids with “anticonvulsant” and “proconvulsant” actions, and hence the net effect of testosterone on neural excitability and seizure activity depends on the levels of distinct testosterone metabolites within the brain (Reddy, 2004a,b). Unlike estradiol, which generally facilitates seizures (Bäckström, 1976; Hom and Buterbaugh, 1986; Buterbaugh, 1989; Woolley, 2000), androstanediol has been shown to produce powerful antiseizure effects (Reddy, 2004b; Kaminski et al., 2005). Testosterone might have a biphasic effect on seizures: proconvulsant at higher doses, anticonvulsant at lower doses. However, testosterone itself has not been reported to improve seizures clinically (Herzog et al., 1998). Reductions of seizures were observed only when testosterone was given together with an estrogen synthesis inhibitor (Herzog et al., 1998), suggesting the estradiol modulation of seizure activity.

In many men with epilepsy, testosterone deficiency is an unusually common clinical observation (Macphee et al., 1988; Herzog, 1991; El-Khayat et al., 2003). TLE surgery has been shown to reduce seizure occurrence and normalize serum androgen concentrations in men with epilepsy (Bauer et al., 2000). Alterations in testosterone levels, therefore, may possibly contribute to exacerbation of seizures. The introduction of finasteride (*Propecia*), which inhibits dihydrotestosterone and androstanediol synthesis, for the treatment of male pattern baldness led to recurrent seizures, which then subsided once the drug was discontinued. Finasteride-induced seizure exacerbation has also been reported recently (Herzog and Frye, 2003). There is a new case report implicating endogenous neurosteroids in TLE (Pugnaghi et al., 2013). Two-week phenytoin treatment has been shown to affect the hippocampal levels of testosterone, cytochrome P450 (CYP) isoforms, and androgen receptor (AR) expression (Meyer et al., 2006). The increased metabolism of testosterone leading to augmented androgen metabolite formation most likely led to enhanced expression of CYP19 and AR in hippocampus, which is a critical area for limbic epileptogenesis.

Aromatase is the key enzyme for the conversion of testosterone to estradiol, a neuroactive steroid that promotes seizures. Aromatase is expressed in discrete areas in the brain such as hippocampus and neocortex that are involved in epileptogenesis. Aromatase inhibitors could decrease brain excitability by decreasing local estradiol levels and therefore, could be beneficial for the treatment of epilepsy (MacLusky et al., 1994). Consequently, aromatase inhibitors have been proposed as a suitable approach to seizure therapy in some men with epilepsy. Some aromatase inhibitors have been tested in men with epilepsy: testolactone, letrozole, and anastrozole. Herzog et al. (1998) tested the efficacy of testosterone and testolactone in men with intractable complex partial seizures. Improvement in seizure control was reportedly achieved with testosterone therapy when testosterone was used along with testolactone. In a case report, letrozole has been shown to improve seizure control in a 61-year-old man with epilepsy (Harden and MacLusky, 2004, 2005). In a pilot study, the safety and efficacy of add-on anastrozole therapy was tested in men with intractable epilepsy. Men with the greatest seizure reduction showed unexpectedly elevated levels in FSH, a pituitary-derived gonadotropin. Hence, the outcome of trials with three distinct aromatase inhibitors – testolactone, letrozole, and anastrozole – suggests a beneficial treatment modality for men with epilepsy (Harden and MacLusky, 2005). Therefore, it is likely that aromatase inhibitors could be potential agents for interruption of proepileptogenic estrogens in the brain.

### ESTROGENS

Although estrogens can affect seizure susceptibility, the role of various estrogens in epileptogenesis is poorly understood. In general, estrogens have proconvulsant and epileptogenic properties in animals and humans (Scharfman and MacLusky, 2006). There are limited studies that support protective effects of estrogens, but it may act as a anticonvulsant under some conditions (Velíšková, 2006). Estradiol has been widely investigated in animal epilepsy models. The effect of estrogens on seizure susceptibility is highly variable and depends on factors such as treatment duration, dosage, hormonal status, and the seizure model (Velíšková, J., and Velísek, 2007). Early studies of estradiol administration to ovariectomized rats revealed proconvulsant effects (Reddy, 2009a). The effect of estrogens on hippocampus seizure susceptibility is controversial (Scharfman and MacLusky, 2006). While estradiol has been shown to be proconvulsant in several studies, there is also evidence that support lack of effect or protective effect of estrogens (Reibel et al., 2000; Velíškováx et al., 2000; Velíšková, J., and Velísek 2007). The effect of circulating estrogens has been studied in female rats with epilepsy (Scharfman et al., 2008, 2009). Epileptic female rats show cyclic increases in epileptiform activity in EEG recordings that coincide with their ovarian cycle, mostly attributable to estrogens.

Estradiol has been known to play a role in the exacerbation of seizures in women with epilepsy (Logothetis et al., 1959; Bäckström, 1976; Jacono and Robinson, 1987). Plasma estradiol levels are found to increase during both the follicular and luteal phase of the normal menstrual cycle. Bäckström (1976) was the first investigator to characterize the relationship between seizures and steroid hormones. In women with epilepsy, a positive correlation between seizure susceptibility and the estrogen-to-progesterone ratio was observed, peaking in the premenstrual and preovulatory periods and declining during the midluteal phase. Logothetis et al. (1959) have demonstrated that intravenous infusions of estrogen were associated with rapid interictal epileptiform activity in women with epilepsy, and seizures were exacerbated when estrogen was given premenstrually. Therefore, it is hypothesized that estrogens may facilitate some forms of catamenial seizures observed during these phases. The periovulatory catamenial exacerbation has been attributed to the midcycle surge of estrogen that is relatively unopposed by progesterone until early luteal phase (Logothetis et al., 1959). An increase in the ratio of estrogen-to-progesterone levels during perimenstrual period might at least partly contribute to the development of perimenstrual catamenial epilepsy (Bonuccelli et al., 1989; Herzog et al., 1997). A recent report from the Nurses’ health study in 114,847 nurses identified key factors associated with seizures in women with epilepsy (Dworetzky et al., 2012). Menstrual irregularity at ages 18–22 years was specifically associated with an increased risk of epilepsy. Menstrual irregularity during follow-up and early age at menarche increased the risk of isolated seizures. Oral contraceptive uses are not associated with isolated seizure or epilepsy.

### GLUCOCORTICOIDS

Pituitary–adrenal hormones have long been known to affect epileptogenesis (Aird and Gordan, 1951; Rose et al., 1979; Weiss et al., 1993; Joëls, 2009; Borekci et al., 2010). Acute stress raises seizure threshold in animals, but chronic stress is known to be a clear risk factor for precipitating seizures in patients with epilepsies. Stress increases plasma and brain concentrations of corticosteroids and neurosteroids. Acute physical or psychological stress causes increased production of hypothalamic corticotrophin releasing hormone (CRH), which is transported via hypophyseal portal system to the pituitary, where it increases both adrenocorticotropic hormone (ACTH) synthesis and secretion. Major physiological effects result from ACTH’s action on adrenal cortex to increase the circulating levels of corticosteroids, principally the glucocorticoid cortisol, and the mineralocorticoid DOC. Cortisol is a major corticosteroid secreted from the adrenal cortex. Cortisol is an excitatory steroid. It elicits proconvulsant and epileptogenic effects (Roberts and Keith, 1995; Joëls, 1997). DOC elicits inhibitory effects and protects against seizures (Reddy, 2003b). An imbalance in cortisol and DOC and other corticosteroids may contribute to susceptibility or resistance to epileptogenesis.

Stress enhances epileptogenesis. In general, chronic or repeated stress has been shown to enhance vulnerability to epileptogenesis in animal models (Joëls, 2009). Corticosterone, the major adrenal steroid in rodents, has been tested extensively in animal models (Weiss et al., 1993; Kumar et al., 2007, 2011; Joëls, 2009; Borekci et al., 2010; Desgent et al., 2012). Prolonged exposure to elevated corticosterone, used as a model of chronic stress, accelerates limbic epileptogenesis (Kumar et al., 2007). Exposure to repeated experimental stress accelerates the development of limbic epileptogenesis, an effect which may be related to elevated corticosterone levels (Jones et al., 2013). Chronic low-dose corticosterone supplementation is shown to enhance epileptogenesis in the rat amygdala kindling model (Kumar et al., 2007). Episodic corticosterone treatment elicits a striking acceleration in kindling epileptogenesis and triggers long-term changes in hippocampal CA1 neurons (Karst et al., 1999). Overall, corticosterone – with other stress hormones – rapidly enhances CA1/CA3 hippocampal activity shortly after stress and could imposes a risk for neuronal injury, such as during epileptic activity. In the hippocampus, stress-induced elevations in neurosteroids promote inhibitory tone mediated through GABA-A receptors. Under conditions of repetitive stress, hormonal influences on the inhibitory tone might diminish and instead, increased excitation become more apparent. In agreement, perinatal stress and elevated corticosteroid levels accelerate epileptogenesis and lower seizure threshold in rodent epilepsy models (Salzberg et al., 2007; Lai et al., 2009; Desgent et al., 2012). Therefore, exposure to stressful events during a critical phase in epileptogenesis could impose lasting deleterious effects on the course of epilepsy.

Deoxycorticosterone, a mineralocorticoid precursor with anesthetic and antiseizure properties, is also produced in the adrenal zona fasciculate. Although the antiseizure properties of DOC in human were first described in 1944 (Aird, 1944; Aird and Gordan, 1951), the mechanisms underlying the brain actions of DOC were only recently identified. The antiseizure activity of DOC requires its enzymatic conversion to 3α,21-dihydroxy-5α-pregnan-20-one (THDOC), a neurosteroid that is a powerful positive allosteric modulator of GABA-A receptors (Reddy, 2003b; **Figure 2**). THDOC is released during physiological stress nearly exclusively from adrenal sources (Purdy et al., 1990; Reddy, 2003b). Plasma and brain levels of THDOC rise rapidly following acute stress (Purdy et al., 1991; Concas et al., 1998; Reddy and Rogawski, 2002). Acute stressors such as swimming, foot shock, or carbon dioxide exposure elicit an increase in allopregnanolone and THDOC concentrations in plasma and in brain (Barbaccia et al., 1996, 1997; Vallée et al., 2000). Stress-induced THDOC and neurosteroids have been demonstrated to elevate seizure threshold (Reddy and Rogawski, 2002) and contributes to neuroprotection (Reddy, 2003b, 2006).

11-Deoxycortisol (pregn-4-ene-17,21-diol-3,20-dione; DC) is an immediate precursor of cortisol. DC acts as a competitive antagonist of glucocorticoid receptor *in vitro*, but is ineffective as a glucocorticoid antagonist *in vivo* due to adrenal 11-hydroxylation (Cutler et al., 1979). Nearly 50 years ago, Heuser and Eidelberg (1961) observed that systemic administration of large doses of DC succinate induces long-lasting seizure activity in rats and cats. The mechanism underlying pro-epileptic properties of DC has been studied recently (Kaminski et al., 2011). DC is capable of inducing long-lasting status epilepticus in rodents that is refractory to several anticonvulsant drugs. In electrophysiological studies, DC is shown to accelerate the decay time of the inhibitory post-synaptic currents mediated by GABA-A receptors in brain slices, indicating that it significantly impedes GABAergic inhibition which may lead to paroxysmal epileptiform network activity and convulsive seizures. Because DC is an endogenous substance, it is suggested to contribute to an increased seizure propensity in some clinical situations. However, the specific role of DC to the pathophysiology of epileptogenesis is remains unclear.

Physical activity has been suggested as a positive disease-modifying factor for preventing or delaying the development of epilepsy. Exercise has beneficial effects on epileptogenesis (Arida et al., 1998, 2007, 2010; Silva de Lacerda et al., 2007). Exercise treatment reduced brain susceptibility in the kindling or the pilocarpine model of epilepsy. Behavioral analysis showed a reduced frequency of seizures during physical exercise program. Metabolic, electrophysiological, and immunohistochemical studies have confirmed the positive influence of exercise on epilepsy (Arida et al., 1999, 2007). Although a variety of factors can contribute to such favorable responses, the mechanisms remain poorly understood. Dendritic plasticity, increased neurogenesis, induction of trophic factors and release of neurosteroids are some factors underlying the inhibitory effects of exercise on epileptogenesis. Enrichment of environment has been shown to delay kindling epileptogenesis in rats (Auvergne et al., 2002). It is likely that endogenous neurosteroids may be involved in the neuroprotective effects of exercise and enriched environment. Thus, prevention of loss of interneurons, reduced GABA-A receptor plasticity, and decrease in axonal sprouting could contribute to the disease-modifying effect of exercise and enriched environment.

Despite acute stress-induced seizure protection in animals (Pericić et al., 2000; Reddy and Rogawski, 2002), patients, and clinicians are not likely to recognize a reduction in seizure frequency associated with stress. It is well known that emotional factors can affect seizure control (Temkin and Davis, 1984). In general, stressful events are associated with more frequent epileptiform spikes and seizures (Frucht et al., 2000). Indeed, stress has been reported to trigger seizure activity in persons with epilepsy (Temkin and Davis, 1984; Frucht et al., 2000). During stressful episodes adrenal hormone levels are expected to fluctuate, possibly affecting epileptogenic events. In agreement with this hypothesis, perinatal stress and elevated steroid levels have been shown accelerate epileptogenesis and lower seizure threshold in various animal models for epilepsy (Joëls, 2009).

Acute stress has anticonvulsant-like effects, while chronic stress is known to induce epileptic seizures. How can such contradictory observations be reconciled? Although the exact pathophysiology of possible seizure facilitation by stress is unknown, there are certainly many neural and endocrine pathways through which stress can alter neuronal excitability and thereby affect seizure susceptibility. The extent of seizure susceptibility during stress might therefore represent a balance between anticonvulsant (e.g., neurosteroids) and proconvulsant factors (e.g., glucocorticoids and CRH). Stress-induced seizures would thus occur when the balance is shifted to favor the proconvulsant factors, surpassing the anticonvulsant action of endogenous neurosteroids (Reddy, 2006). Although little is known regarding proconvulsant factors, stress can increase brain levels of “proconvulsant” sulfated neurosteroids such as pregnenolone sulfate (PS) and dehydroepiandrosterone sulfate (DHEAS; see Role of Neurosteroids in Epileptogenesis). Additionally, repeated episodes of stress and neurosteroid release might lead to a sort of neurosteroid withdrawal-induced hyperexcitable state (Reddy et al., 2001, 2012) and could predispose patients to stress-induced seizures. Nevertheless, alleviating the effects of stress by pharmacological interventions may help reduce the epileptogenicity in people with risk factors for epilepsy.

## ROLE OF NEUROSTEROIDS IN EPILEPTOGENESIS

Neurosteroids are steroids synthesized within the brain with unconventional rapid effects on neuronal excitability. It is well known that steroid hormones such as progesterone and DOC can exert anticonvulsant actions (Selye, 1941; Clarke et al., 1973). The anticonvulsant properties of progesterone and DOC are predominantly due to their conversion in the brain to neurosteroids allopregnanolone (3α-hydroxy-5α-pregnane-20-one, AP) and allotetrahydrodeoxycorticosterone (THDOC), respectively (Reddy, 2003a; Reddy et al., 2004; **Figure 2**). A variety of neurosteroids are known to be synthesized in the brain (Baulieu, 1981; Kulkarni and Reddy, 1995). The most widely studied are AP, THDOC, and androstanediol. These neurosteroids are produced via sequential A-ring reduction of the steroid hormones by 5α-reductase and 3α-hydroxysteroid-oxidoreductase (3α-HSOR) isoenzymes (Reddy, 2009a). The androgenic neurosteroid androstanediol (5α-androstan-3α,17β-diol; **Figure 2**) is synthesized from testosterone (Reddy, 2004a,b).

In the periphery, the steroid precursors are mainly synthesized in the gonads, adrenal gland, and feto-placental unit, but synthesis of these neurosteroids likely occurs in the brain from cholesterol or from peripherally derived intermediates. Since neurosteroids are highly lipophilic and can readily cross the blood–brain barrier, neurosteroids synthesized in peripheral tissues accumulate in the brain (Reddy and Rogawski, 2010). Recent evidence indicates that neurosteroids are present mainly in principal neurons in many brain regions that are relevant to focal epilepsies, including the hippocampus and neocortex (Agís-Balboa et al., 2006; Saalmann et al., 2007; Do Rego et al., 2009). The biosynthesis of neurosteroids is controlled by the translocator protein (18 kDa; TSPO), formerly called peripheral or mitochondrial benzodiazepine receptor (Rupprecht et al., 2009, 2010). Activation of TSPO by endogenous signals and ligands facilitates the intramitochondrial flux of cholesterol and thereby promotes neurosteroid synthesis. It is suggested that TSPO ligands might be an alternative approach for neurosteroid therapeutics (Nothdurfter et al., 2011). Currently, synthetic analogs of endogenous neurosteroids are under clinical trial for treatment of epilepsy (Reddy and Rogawski, 2010, 2012).

### POTENTIATION OF PHASIC AND TONIC INHIBITION

**FIGURE 3:**
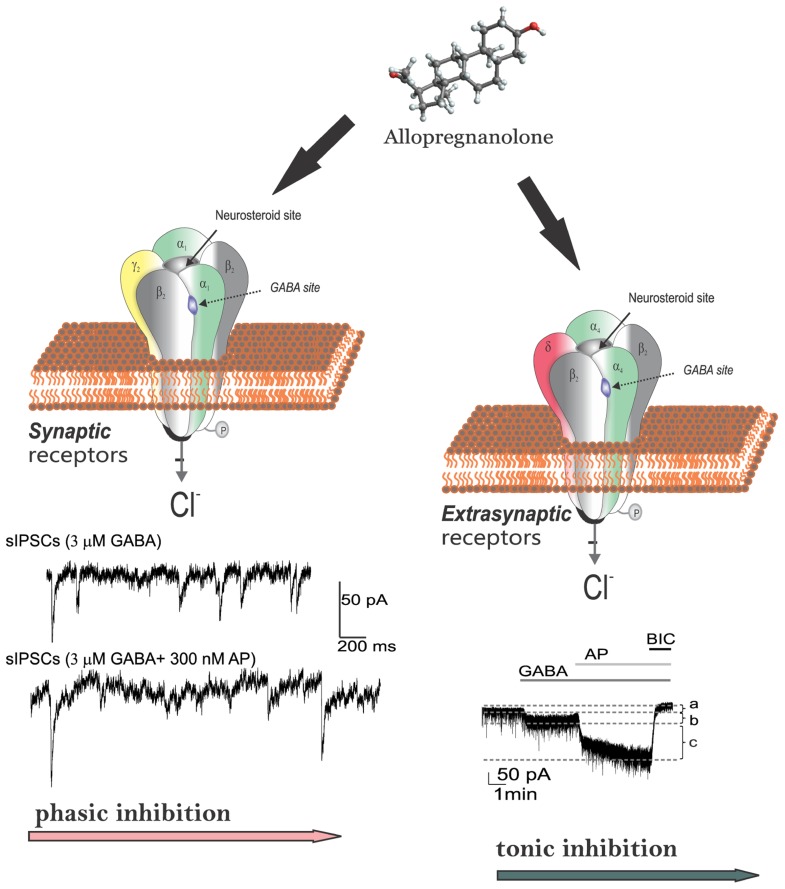
**Neurosteroid modulation of GABA-A receptors in the brain.** Allopregnanolone and related neurosteroids binds and enhances GABA-A receptor-mediated inhibition in the brain. GABA-A receptors are pentameric with five protein subunits that form the chloride ion channel pore. Neurosteroids directly binds to the “neurosteroid binding sites” and potentiate the GABA-gated chloride currents. The neurosteroid binding sites are distinct from sites for GABA, benzodiazepines, and barbiturates. There are two types of GABA-A receptors with different functions. Post-synaptic GABA-A receptors, which are pentameric chloride channels composed of 2α2βγ subunits, mediate the phasic portion of GABAergic inhibition, while extrasynaptic GABA-A receptors, pentamers composed of 2α2βδ subunits, primarily contribute to tonic inhibition in the hippocampus. Neurosteroids activate both synaptic and extrasynaptic receptors and enhance the phasic and tonic inhibition, and thereby promote maximal network inhibition in the brain.

Neurosteroids rapidly alter neuronal excitability through direct interaction with GABA-A receptors (Harrison and Simmonds, 1984; Majewska et al., 1986; Harrison et al., 1987; Gee et al., 1988; Purdy et al., 1990; Hosie et al., 2007, 2009), which are the major receptors for the inhibitory neurotransmitter GABA. Activation of the GABA-A receptor by various ligands leads to an influx of chloride ions and to a hyperpolarization of the membrane that dampens the excitability. Allopregnanolone and other structurally related neurosteroids act as positive allosteric modulators and direct activators of GABA-A receptors (**Figure 3**). At low concentrations, neurosteroids potentiate GABA-A receptor currents, whereas at higher concentrations, they directly activate the receptor (Harrison et al., 1987; Reddy and Rogawski, 2002). Like barbiturates, neurosteroid enhancement of GABA-A receptors occurs through increases in both the channel open frequency and channel open duration (Twyman and Macdonald, 1992; Lambert et al., 2009; Ramakrishnan and Hess, 2010). The GABA-A receptor is a pentamer consisting of five subunits that form a chloride channel. Sixteen subunits (α1-6, β1-3, γ1-3, δ, ε, θ, and π subunits) have been identified so far. The GABA site is located at the interface between α and β subunits. Benzodiazepines bind at the interface between α and γ subunits and they interact with subunit combinations α1,2,3,5β2γ2.

Activation of GABA-A receptors produces two forms of inhibition: phasic inhibition generated by the rapid, transient activation of synaptic GABA-A receptors by presynaptic GABA release, and tonic inhibition generated by the persistent activation of extrasynaptic GABA-A receptors, which can detect extracellular GABA (**Figure 3**). There are major differences between synaptic and extrasynaptic GABA-A receptors (**Table 3**). The extrasynaptic GABA-A receptors are distributed within the hippocampus (α4βδ, α5βδ, or α1βδ), neocortex (α4βδ, α5βδ), thalamus (α4βδ), striatum (α4βδ), hypothalamus (α4βδ), and cerebellum (α6βδ). Although GABA activates synaptic (γ2-containing) GABA-A receptors with high-efficacy, GABA activation of the extrasynaptic (δ-containing) GABA-A receptors are limited to low-efficacy activity characterized by minimal desensitization and brief openings. Such tonic currents are particularly evident in dentate granule cells, which play a major role in hippocampus excitability. The high sensitivity of δ-containing receptor channels to neurosteroid modulation may be dependent on the δ-subunit or the low-efficacy channel function that it confers. There is evidence that neurosteroids preferentially enhance low-efficacy GABA-A receptor activity independent of subunit composition (Bianchi and Macdonald, 2003).

**Table 3:** An overview of synaptic (αβγ2-containing) and extrasynaptic (αβδ-containing) GABA-A receptors in the brain.

Synaptic GABA-A receptors	Extrasynaptic GABA-A receptors
Pentameric chloride ion channels	Pentameric chloride ion channels
Contributes to phasic inhibition	Contributes to tonic inhibitiion
Low GABA affinity	High GABA affinity
High GABA efficacy	Low GABA efficacy
Pronounced desensitization	Moderate or low desensitization
Mainly synaptic localization	Perisynaptic and extrasynaptic sites
Benzodiazepine sensitive	Benzodiazepine insensitive
Potentiated by neurosteroids	Highly potentiated by neurosteroids
Not blocked by low [Zn^2+^]	Blocked by low [Zn^2+^]
Distributed widely within the brain: cortex, hippocampus, amygdala, limbic structures, thalamus, hypothalamus, cerebellum	Selective distribution in few brain regions: hippocampus, neocortex, thalamus, hypothalamus, cerebellum

The effect of neurosteroids on GABA-A receptors occurs by binding to discrete sites on the receptor–channel complex that are located within the transmembrane domains of the α- and β-subunits (Hosie et al., 2006, 2007), which they access by lateral membrane diffusion (Chisari et al., 2009, 2010; **Figure 3**). The binding sites for neurosteroids are distinct from the recognition sites for GABA, benzodiazepines, and barbiturates (Hosie et al., 2009). Androgenic neurosteroids such as androstanediol may interact with these sites, and a recent study indicates that this agent is a positive allosteric modulator of GABA-A receptors (Reddy and Jian, 2010). Although neurosteroids act on all GABA-A-receptor isoforms, they have large effects on extrasynaptic δ-subunit containing GABA-A receptors that mediate tonic currents (Belelli et al., 2002; Wohlfarth et al., 2002). The potentiation of δ-subunit-containing receptors by THDOC and other neurosteroids is selective for channels with low-efficacy gating characteristics marked by brief bursts and channel openings in conditions of both low and high GABA concentrations, and neurosteroids can thereby preferentially increase the efficacy of these receptors based on pharmacokinetics which are not yet fully understood (Bianchi and Macdonald, 2003). Neurosteroids therefore markedly enhance the current generated by δ-subunit-containing receptors even in the presence of saturating GABA concentrations. Consequently, GABA-A receptors that contain the δ-subunit are highly sensitive to neurosteroid potentiation and mice lacking δ-subunits show drastically reduced sensitivity to neurosteroids (Mihalek et al., 1999; Spigelman et al., 2002). Tonic current causes a steady inhibition of neurons and reduces their excitability. Neurosteroids therefore could play a role in setting the level of excitability by potentiation of tonic inhibition during seizures when ambient GABA rises (Stell et al., 2003).

The pharmacological profile of major neurosteroids is outlined in **Table 4**. Allopregnanolone-like neurosteroids are powerful antiseizure agents. Exogenously administered neurosteroids exhibit broad-spectrum anticonvulsant effects in diverse rodent seizure models (Reddy, 2010). Neurosteroids protect against seizures induced by GABA-A receptor antagonists, including pentylenetetrazol and bicuculline, and are effective against pilocarpine-induced limbic seizures and seizures in kindled animals (Belelli et al., 1989; Kokate et al., 1994; Frye, 1995; Wieland et al., 1995; Reddy and Rogawski, 2001, 2010; Kaminski et al., 2004, 2005; Reddy et al., 2004). Like other GABAergic agents, they may exacerbate generalized absence seizures (Snead, 1998; Citraro et al., 2006). The potencies of neurosteroids in models where they confer seizure protection vary largely in accordance with their activities as positive allosteric modulators of GABA-A receptors (Reddy, 2004a,b; Kaminski et al., 2005). Like other GABAergic agents, neurosteroids are inactive or only weakly active against seizures elicited by maximal electroshock. Neurosteroids are highly active in the 6-Hz model, a better paradigm in which limbic-like seizures are induced by electrical stimulation of lower frequency and longer duration than in the maximal electroshock test (Kaminski et al., 2004). Androstanediol, but not its 3β-epimer, produced a dose-dependent suppression of behavioral and electrographic seizures in the mouse hippocampus kindling (Reddy and Jian, 2010). In addition, neurosteroids are also highly effective in suppressing seizures due to withdrawal of GABA-A receptor modulators including neurosteroids and benzodiazepines, as well as other types of agents such as ethanol and cocaine (Devaud et al., 1996; Tsuda et al., 1997; Reddy and Rogawski, 2001; Gangisetty and Reddy, 2010). In contrast to benzodiazepines, where utility in the chronic treatment of epilepsy is limited by tolerance, anticonvulsant tolerance is not evident with neurosteroids (Kokate et al., 1998; Reddy and Rogawski, 2000), which indicate that neurosteroids are more effective than benzodiazepines for long-term treatment. Novel therapeutic approaches are being developed based on the emerging information on neurosteroid interaction with GABA-A receptors (Reddy and Rogawski, 2009; Murashima and Yoshii, 2010).

**Table 4:** Pharmacological profile of major neurosteroids in animal models.

Seizure model	Allopreg-nanolone	THDOC	Andro-stanediol
**Kindling models**
Hippocampus kindling	3.5	ND	50 (36-64)
Amygdala kindling	14 (8–23)	15 (10–30)	ND
**Chemoconvulsant models**
Pentylenetetrazol	12 (10–15)	19 (77–122)	40 (27–60)
Bicuculline	12 (10–15)	12 (10–15)	44 (24–81)
Picrotoxin	10 (5–19)	10 (5–19)	39 (21–74)
*N*-Methyl-D-aspartate	>40	>40	>200
Kainic acid	>40	>40	>200
4-Aminopyridine	>40	>40	>200
**Electroshock models**
Maximal electroshock	29 (19–44)	48 (35–66)	ND
6-Hz stimulation	14 (10–19)	ND	ND
**Status epilepticus models**
Pilocarpine	7 (4–13)	7 (4–13)	81 (45–133)

*The profile of neurosteroids is expressed in terms of ED_50_, which is the dose in mg/kg producing seizure protection in 50% of animals. Values in parentheses are 95% confidence limits. ND, not determined.*

### ANTIEPILEPTOGENIC ACTIVITY

Neurosteroids may play a role in chronic epilepsy. Neurosteroid modulation of tonic activation of extrasynaptic GABA-A receptors can regulate excitability during epileptogenicity. Given the complex plasticity in GABA-A receptors in epilepsy, it is difficult to predict the functional outcome of altered subunit compositions. A consistent finding from studies that have used various models of chronic epilepsy is that tonic conductances are largely preserved in epileptic brain around the time when synaptic inhibition is reduced (Mtchedlishvili et al., 2001; Sun et al., 2007; Zhang et al., 2007). Studies in a status epilepticus model of TLE have shown a striking reduction in δ-subunit containing GABA-A receptors in the dentate gyrus (Peng et al., 2004; Zhang et al., 2007), suggesting that neurosteroid effects on non-synaptic GABA-A receptors may be reduced. There was a compensatory increase in γ2-subunit, so that tonic inhibition is preserved though the efficacy of THDOC in modulating tonic current is decreased. In addition, neurosteroid modulation of synaptic currents is diminished in dentate gyrus granule cells and α4-subunit-containing receptors are expressed at synaptic sites (Sun et al., 2007). All of these changes may exacerbate seizures in epileptic animals and reduce the potency but not efficacy of endogenous neurosteroids. The expression of neurosteroidogenic enzymes such as P450scc and 3α-HSOR appears to be elevated in the hippocampus in animals and human subjects affected by TLE (Stoffel-Wagner et al., 2000; VMDB Report, 2003; Biagini et al., 2009). If local neurosteroidogenesis is enhanced, this may in part counteract the epileptogenesis-induced changes.

**FIGURE 4:**
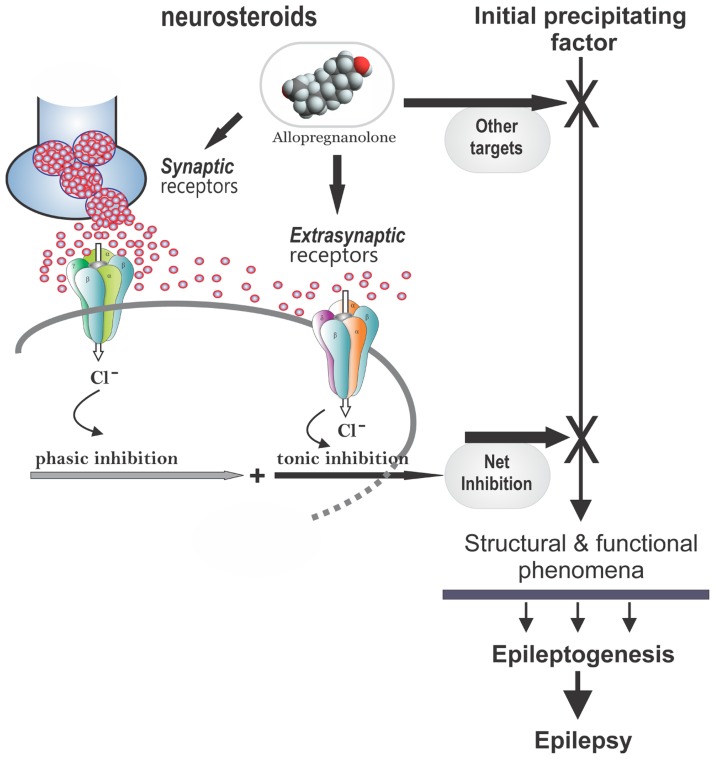
**Potential molecular mechanisms of neurosteroid interruption of epileptogenesis.** In the brain, allopregnanolone and related neurosteroids may retard epileptogenesis by the interruption of one or more of the pathways leading to development of epilepsy, which generally occurs following an initial precipitating event. Neurosteroids such as allopregnanolone binds to synaptic and extrasynaptic GABA-A receptors and enhances phasic and tonic inhibition within the brain, and thereby may affect epileptogenesis. Other potential mechanisms include modulation of neuroinflammation and neurogenesis in the brain.

There is emerging evidence that endogenous neurosteroids play a role in regulating epileptogenesis (Edwards et al., 2001; Biagini et al., 2006, 2009, 2010; Reddy et al., 2010; **Figure 4**). Using the kindling model, we demonstrated that the development and persistence of limbic epileptogenesis are impaired in mice lacking PRs (Reddy and Mohan, 2011). To explore mechanisms underlying the observed seizure resistance, we investigated the role of neurosteroids using finasteride, a 5α-reductase inhibitor that blocks the synthesis of progesterone-derived neurosteroids. We determined the rate of rapid kindling in both control animals and those which had received injections of progesterone with or without concurrent finasteride treatment (Reddy and Mohan, 2011). Progesterone produced a significant delay in the rate of kindling and pretreatment with finasteride blocked progesterone’s inhibition of kindling epileptogenesis (Reddy and Ramanathan, 2012). These findings are consistent with a contributory role of neurosteroids in limbic epileptogenesis. Thus, it is possible that inhibition of neurosteroids could incite mechanisms that may promote epileptogenesis.

The P450sc is a critical enzyme for the biosynthesis of neurosteroids (**Figure 2**). It is present in neurons, oligodendrocytes, astrocytes, and activated microglia. Following pilocarpine-induced status epilepticus in the rat, the neurosteroidogenic enzyme P450scc is upregulated for several weeks, suggesting that it may be associated with promotion of neurosteroidogenesis (Biagini et al., 2009). Ordinarily, rats develop spontaneous recurrent seizures following a latent period of similar duration to the period during which P450scc is elevated. The role of neurosteroids in delaying seizure onset in the pilocarpine model is confirmed using finasteride, which can exacerbate seizures by inhibition of neurosteroid synthesis. Inhibiting neurosteroid synthesis with finasteride accelerated the onset of spontaneous recurrent seizures (Biagini et al., 2006), suggesting that endogenous neurosteroids play a role in restraining epileptogenesis, or at least act to inhibit the expression of seizures.

The development of epilepsy is linked to complex alterations in neuroplastic mechanisms. Dysregulation of neurosteroid synthesis may also play a role. This premise is being tested in various epileptogenic models (Reddy and Mohan, 2011). We investigated the role of the prototype endogenous neurosteroid allopregnanolone in controlling limbic epileptogenesis. Treatment with finasteride, a neurosteroid synthesis inhibitor, resulted in a significant increase in epileptogenesis in the hippocampus kindling model (Ramanathan and Reddy, 2011). Exogenous administration of allopregnanolone, at doses that produce levels similar to gonadotropins, markedly inhibited epileptogenesis. In female epilepsy rats, finasteride treatment exacerbates seizure frequency (Lawrence et al., 2010). Neurosteroid-mediated increase in tonic inhibition in the hippocampus could inhibit the spread of the seizure discharge from the hippocampal focus and thereby suppress the rate of development of behavioral kindled seizure activity without affecting the focal electrographic discharges (**Figure 4**). The exact mechanisms are unclear. Increased tonic inhibition by allopregnanolone is shown to impair the NMDA (*N*-methyl-D-aspartate) receptor-mediated excitability in the hippocampus (Shen et al., 2010). It is likely that such a mechanism may underlie the progesterone’s disease-modifying effects in the kindling model. Based on these pilot studies, it is suggested that augmentation of neurosteroid synthesis may represent a unique strategy for preventing or retarding epileptogenesis.

### PROCONVULSANT AND EPILEPTOGENIC ACTIVITY

Neurosteroids that are sulfated at C3 have inhibitory actions on GABA-A receptors (Park-Chung et al., 1999). PS and DHEAS block GABA-A receptors at low micromolar concentrations (Majewska, 1992). These “sulfated steroids” act as non-competitive antagonists of the GABA-A receptor by interacting with a site that is distinct from that of neurosteroids such as allopregnanolone and THDOC (Majewska and Schwartz, 1987; Majewska et al., 1990; Park-Chung et al., 1999). The steroid negative modulatory action on GABA-A receptors occurs through a reduction in channel opening frequency, although the precise mechanism of block is not well understood (Mienville and Vicini, 1989; Akk et al., 2001). PS has been shown to possess agonistic actions on NMDA receptors (Wu et al., 1991) and presynaptic sites (Mtchedlishvili and Kapur, 2003). Given their abundance in brain, it seems reasonable that PS and DHEAS could function as endogenous neuromodulators. In contrast to allopregnanolone, sulfated neurosteroids PS and DHEAS exert proconvulsant or convulsant actions (Reddy and Kulkarni, 1998; Kokate et al., 1999b). Direct injection of PS to rodent brain elicits seizures which increase in severity and frequency with time and eventually progress to status epilepticus, tonic hindlimb extension, and death of the animals (Kokate et al., 1999b; Williamson et al., 2004). However, systemic administration of PS does not induce overt seizure activity (Kokate et al., 1999b). The pharmacology of PS seizures is poorly understood, but both clonazepam and allopregnanolone can effectively protect against PS-induced seizure activity (Kokate et al., 1999b). The proconvulsant effect of DHEAS is reduced by progesterone, an intermediate precursor of neurosteroid synthesis. Moreover, exogenous application or endogenous stimulation of DHEAS modulates hippocampal GABA inhibition possibly by entraining hippocampal neurons to theta rhythm (Steffensen, 1995), suggesting a potential physiological relevance of the proconvulsant effects of DHEAS in animals.

## GENDER DIFFERENCES IN EPILEPSY AND NEUROSTEROIDS

Epilepsy shows sex differences in incidence, progression, and severity, as well as in responsiveness to therapy. The incidence of epilepsy is generally higher in males than in females. More women than men are diagnosed with idiopathic generalized epilepsy, but localization-related symptomatic epilepsies are more frequent in men, and cryptogenic localization-related epilepsies are more frequent in women (Hauser, 1997; Christensen et al., 2005). Sex differences have been described in patients with TLE, with respect to distinct regional distribution of brain dysfunction during interictal periods as well as to the extent of neuronal damage. Women tend to have less structural atrophy than men, regardless of the seizure rate. During brain development, sex hormones have organizational effects leading to permanent differences between males and females in distinct brain regions (Velíšková, 2009). However, the precise mechanisms underlying the sex-dependent differentiation of the specific neuronal circuits, particularly brain regions involved in seizure control, are not clear. Many factors are involved in determining sex differences in seizure susceptibility, including the presence of sexual dimorphism in brain structures involved in seizure generation and control, in regional connectivity, sensitivity of neurotransmitter systems, receptor distribution, and dependence on hormonal milieu and on changes in sex hormone levels during the life span.

Neurosteroids exhibit a strong gender differences in their pharmacological effects with more potency in females than males (Reddy, 2009b). Steroid hormones such as progesterone and testosterone play a key role in the gender-related differences in susceptibility to seizures. However, the precise mechanism underlying such sexual dimorphism is obscure. Many of the biological actions of steroid hormones are mediated through intracellular receptors. Studies have suggested that these sex differences in seizure sensitivity are due to gender-specific distribution of steroid hormones or other sexually dimorphic characteristics in specific brain areas relevant to epilepsy. For example, estradiol reduces seizure-induced hippocampal injury in ovariectomized female but not in male rats, suggesting that the effects of estradiol on seizure threshold and damage may be determined by sex-related differences in the hormonal environment. Neurosteroids may play a key role in gender-related differences in seizure susceptibility (Reddy, 2009b). Both progesterone and allopregnanolone protect against experimental seizures in both male and female mice lacking PRs (Reddy et al., 2004). However, female mice exhibit significantly enhanced sensitivity to the protective activity of allopregnanolone as compared to males. In the pilocarpine seizure test, androstanediol has similar increased potency in female mice, which is not related to differences in pharmacokinetics of this neurosteroid. These results underscore the possible role of endogenous neurosteroids in gender-related differences in seizure susceptibility and protection.

## POTENTIAL SIDE EFFECTS OF NEUROSTEROIDS

Steroid hormones such as progesterone and DOC have long been known to have sedative, anesthetic, and antiseizure properties (Aird, 1944; Gyermek et al., 1967; Green et al., 1978). Studies during the past three decades have uncovered that neurosteroids mediate such rapid effects of steroid hormones in the brain (Reddy et al., 2004, 2005). Unlike steroid hormones, the acute effects of neurosteroids are not related to interactions with classical steroid hormone receptors that regulate gene transcription. Moreover, neurosteroids are not themselves active at intracellular steroid receptors. They modulate brain excitability primarily by interaction with neuronal membrane receptors and ion channels, principally GABA-A receptors. Therefore, like other GABAergic agents, neurosteroids have sedative and anxiolytic properties (Reddy, 2003a). At high doses, neurosteroids cause anesthetic effects. This feature is exemplified with alphaxolone, which was introduced as intravenous anesthetic in 1970s. It was withdrawn later from the market due to toxicity of the solvent used for formulation of this synthetic neurosteroid. Although natural neurosteroids can be used for therapeutic purpose in patients with epilepsy, certain obstacles prevent the clinical use of endogenous neurosteroids. First, natural neurosteroids such as allopregnanolone have low bioavailability because they are rapidly inactivated and eliminated by glucuronide or sulfate conjugation at the 3α-hydroxyl group. Secondly, the 3α-hydroxyl group of allopregnanolone may undergo oxidation to the ketone, restoring activity at steroid hormone receptors (Rupprecht et al., 1993). Synthetic neurosteroids, which are devoid of such hormonal actions, could provide a rational alternative approach to therapy (Reddy and Kulkarni, 2000). Recently, a number of synthetic analogs of allopregnanolone and other neurosteroids are tested in animals and human trials. Ganaxolone, the 3β-methyl analog of allopregnanolone, is a synthetic neurosteroid analog that overcomes these limitations (Carter et al., 1997). Results from the clinical trials of ganaxolone demonstrated a benign side effect profile (Reddy and Woodward, 2004). More than 900 subjects (adults and children) have received this neurosteroid in Phase 1 and 2 trials. Overall, the drug is safe and well tolerated. The most common side effect is reversible dose-related sedation. Other adverse events reported by few subjects include dizziness, fatigue, and somnolence, which indicate its GABAergic effects.

## CONCLUSION

The major part of the pathophysiology of epilepsy is epileptogenesis, whereby a normal brain becomes progressively epileptic because of injury factors. Despite increased scientific awareness, there is a large gap in our understanding of epileptogenesis and many questions remain unanswered regarding the cellular and molecular mechanisms underlying the “irreversible conversion” of a normal brain into epileptic brain predisposed to recurrent seizures. Such gaps in our knowledge about epilepsy’s risk factors, comorbidities, and outcomes limit the ability of clinical programs to prevent epilepsy and its consequences. Since the Curing Epilepsy conference in 2000, there has been more focused research on optimizing approaches in preventing epilepsy and there is emphasis on several translation models. Reduction in neuroinflammation and neurodegeneration is a widely targeted approach for curing epilepsy. Despite intense search for drugs that interrupt epileptogenesis, presently there is no FDA-approved drug available for prevention of epilepsy development in patients at risk. It is essential to find a lead target, such as a receptor or signaling pathway that is crucial for the progression of epileptogenesis that can be disrupted by pharmacological agents to prevent or retard epilepsy.

A variety of pharmacological agents have been tested in animal models of epileptogenesis and in clinical trials in patients with a risk factor for epilepsy. However, the outcomes are not highly promising. The pipeline of new drugs for clinical development is very limited. A summary of agents that are currently in development for epilepsy is listed in **Table 5**. Steroid hormones, especially corticosteroids, estrogen, and progesterone play an important role in epilepsy. Repetitive stress and corticosteroids increases the risk of epilepsy. There is emerging evidence that neurosteroids may play a role in limbic epileptogenesis. Neurosteroids that enhance the GABAergic inhibition within the brain are potent anticonvulsants and they regulate neural excitability networks by enhancing the phasic and tonic inhibition in the hippocampus, a critical region involved in the TLE. Tonic inhibition is therefore an attractive target for antiepileptogenic drugs (**Figure 4**). The effects of stress and hormonal changes on neuronal excitability are most likely mediated by neurosteroids. Therefore, menstrual, gonadal, and stress-related fluctuations in neurosteroids or dysfunction in their synthesis can alter epileptogenesis in people at risk for epilepsy. As pleotropic agents, steroid hormones can modify, interrupt or reverse the epileptogenic process, such as cell loss, neuroinflammation, neurogenesis, astrogliosis, and axonal sprouting.

**Table 5:** Pipeline of new drugs for epilepsy under development.

Agent	Pharmacological profile
BGG492 (Novartis)	A competitive AMPA/kainate receptor antagonist
Brivaracetam (UCB)	A novel high-affinity synaptic vesicle protein 2A (SV2A) ligand
CPP-115 (Catalyst)	A GABA transaminase inhibitor (vigabatrin derivative)
ICA-105665 (Pfizer)	A highly selective opener of neuronal Kv7 (KCNQ) potassium channels
T2000 (Taro)	A non-sedating barbiturate (prodrug of diphenylbarbituric acid)
Tonabersat (Upsher-Smith)	A novel mechanism of uncoupling of neuronal gap junctions
UCB-0942 (UCB)	A new pre-and post-synaptic inhibitor
VX765 (Vertex)	A selective inhibitor of interleukin converting enzyme
YKP3089 (SK Life)	Novel mechanism of action
2-Deoxy-D-glucose (NeuroGenomeX)	A glucose analog and glycolytic inhibitor
Ganaxolone (Marinus)	A synthetic neurosteroid and GABA-A receptor modulator
Imepitoin (BI)	A low-affinity partial agonist at the benzodiazepine site of the GABA-A receptor
NAX 810-2 (NeuroAdjuvants)	Galanin receptor GalR1 and GalR2 agonist
Valnoctamide (Hebrew Univ)	Valproic acid second generation derivative

## Conflict of Interest Statement

The author declares that the research was conducted in the absence of any commercial or financial relationships that could be construed as a potential conflict of interest.

## References

[B1] Acharya M. M. Hattiangady B. Shetty A. K. (2008). Progress in neuroprotective strategies for preventing epilepsy. *Prog. Neurobiol.* 84 363–404 10.1016/j.pneurobio.2007.10.010 18207302 PMC2441599

[B2] Agís-Balboa R. C. Pinna G. Zhubi A. Maloku E. Veldic M. Costa E. (2006). Characterization of brain neurons that express enzymes mediating neurosteroid biosynthesis. *Proc. Natl. Acad. Sci. U.S.A.* 103 14602– 14607 10.1073/pnas.0606544103 16984997 PMC1600006

[B3] Aird R. B. (1944). The effect of desoxycorticosterone in epilepsy. *J. Nerv. Ment. Dis.* 99 501–510 10.1097/00005053-194405000-00005

[B4] Aird R. B. Gordan G. S. (1951). Anticonvulsive properties of desoxycorticosterone. *J. Am. Med. Assn.* 145 715–719 10.1001/jama.1951.02920280027006 14803247

[B5] Akk G. Bracamontes J. Steinbach J. H. (2001). Pregnenolone sulfate block of GABAA receptors: mechanism and involvement of a residue in the M2 region of the subunit. *J. Physiol.* 532 673–684 10.1111/j.1469-7793.2001.0673e.x 11313438 PMC2278584

[B6] Arida R. M. de Jesus Vieira A. Cavalheiro E. A. (1998). Effect of physical exercise on kindling development. *Epilepsy Res.* 30 127–132 10.1016/S0920-1211(97)00102-2 9600544

[B7] Arida R. M. Scorza F. A. Cavalheiro E. A. (2010). Favorable effects of physical activity for recovery in temporal lobe epilepsy. *Epilepsia* 51(Suppl. 3) 76–79 10.1111/j.1528-1167.2010.02615.x 20618406

[B8] Arida R. M. Scorza F. A. de Lacerda A. F. Gomes da Silva S. Cavalheiro E. A. (2007). Physical training in developing rats does not influence the kindling development in the adult life. *Physiol. Behav.* 90 629–633 10.1016/j.physbeh.2006.11.016 17196228

[B9] Arida R. M. Scorza F. A. dos Santos N. F. Peres C. A. Cavalheiro E. A. (1999). Effect of physical exercise on seizure occurrence in a model of temporal lobe epilepsy in rats. *Epilepsy Res.* 37 45–52 10.1016/S0920-1211(99)00032-7 10515174

[B10] Austin J. K. Hesdorffer D. C. Liverman C. T Schultz A. M Testimony Group (2012). Testimonies submitted for the Institute of Medicine report: epilepsy across the spectrum: promoting health and understanding. *Epilepsy Behav*. 25 634–661 10.1016/j.yebeh.2012.10.003 23158808

[B11] Auvergne R. Leré C. El Bahh B. Arthaud S. Lespinet V. Rougier A. (2002). Delayed kindling epileptogenesis and increased neurogenesis in adult rats housed in an enriched environment. *Brain Res.* 954 277–285 12414110 10.1016/s0006-8993(02)03355-3

[B12] Bäckström T. (1976). Epileptic seizures in women related to plasma estrogen and progesterone during the menstrual cycle. *Acta Neurol. Scand.* 54 321–347 973554 10.1111/j.1600-0404.1976.tb04363.x

[B13] Bäckström T. Zetterlund B. Blom S. Romano M. (1984). Effect of intravenous progesterone infusions on the epileptic discharge frequency in women with partial epilepsy. *Acta Neurol. Scand.* 69 240–248 6430018 10.1111/j.1600-0404.1984.tb07807.x

[B14] Barbaccia M. L. Roscetti G. Trabucchi M. Mostallino M. C. Concas A. Purdy R. H. (1996). Time-dependent changes in rat brain neuroactive steroid concentrations and GABA-A receptor function after acute stress. *Neuroendocrinology* 63 166–172 10.1159/000126953 9053781

[B15] Barbaccia M. L. Roscetti G. Trabucchi M. Purdy R. H. Mostallino M. C. Concas A. (1997). The effects of inhibitors of GABAergic transmission and stress on brain and plasma allopregnanolone concentrations. *Br. J. Pharmacol.* 120 1582–1588 10.1038/sj.bjp.0701046 9113382 PMC1564614

[B16] Bauer J. Stoffel-Wagner B. Flügel D. Kluge M. Schramm J. Bidlingmaier F. (2000). Serum androgens return to normal after temporal lobe epilepsy surgery in men. *Neurology* 55 820–824 10994003 10.1212/wnl.55.6.820

[B17] Baulieu E. E. (1981). “Steroid hormones in the brain: several mechanisms,” in *Steroid Hormone Regulation of the Brain* eds Fuxe F. Gustafsson J. A. Wetterberg L. (Oxford: Pergamon Press) 3–14

[B18] Belelli D. Bolger M. B. Gee K. W. (1989). Anticonvulsant profile of the progesterone metabolite 5 α-pregnan-3 α-ol-20-one. *Eur. J. Pharmacol.* 166 325–329 10.1016/0014-2999(89)90077-0 2792198

[B19] Belelli D. Casula A. Ling A. Lambert J. J. (2002). The influence of subunit composition on the interaction of neurosteroids with GABAA receptors. *Neuropharmacology* 43 651–661 10.1016/S0028-3908(02)00172-7 12367610

[B20] Biagini G. Baldelli E. Longo D. Pradelli L. Zini I. Rogawski M. A. (2006). Endogenous neurosteroids modulate epileptogenesis in a model of temporal lobe epilepsy. *Exp. Neurol.* 201 519–524 10.1016/j.expneurol.2006.04.029 16780839

[B21] Biagini G. Longo D. Baldelli E. Zoli M. Rogawski M. A. Bertazzoni G. (2009). Neurosteroids and epileptogenesis in the pilocarpine model: evidence for a relationship between P450scc induction and length of the latent period. *Epilepsia* 50(Suppl. 1) 53–58 10.1111/j.1528-1167.2008.01971.x 19125849 PMC4873280

[B22] Biagini G. Panuccio G. Avoli M. (2010). Neurosteroids and epilepsy. *Curr. Opin. Neurol.* 23 170–176 10.1097/WCO.0b013e32833735cf 20160650 PMC4873277

[B23] Bianchi M. T. Macdonald R. L. (2003). Neurosteroids shift partial agonist activation of GABA-A receptor channels from low- to high-efficacy gating patterns. *J. Neurosci.* 23 10934–10943 14645489 10.1523/JNEUROSCI.23-34-10934.2003 PMC6740972

[B24] Bonuccelli U. Melis G. B. Paoletti A. M. Fioretti P. Murri L. Muratorio A. (1989). Unbalanced progesterone and estradiol secretion in catamenial epilepsy. *Epilepsy Res.* 3 100–106 10.1016/0920-1211(89)90037-5 2651113

[B25] Borekci B. Ingec M. Yilmaz M. Kukula O. Karaca M. Hacimuftuoglu A. (2010). Effects of female sex hormones on caffeine-induced epileptiform activity in rats. *Gynecol. Endocrinol.* 26 366–371 10.3109/09513590903511513 20063987

[B26] Brinton R. D. Thompson R. F. Foy M. R. Baudry M. Wang J. Finch C. E. (2008). Progesterone receptors: form and function in brain. *Front. Neuroendocrinol.* 29:313–339 10.1016/j.yfrne.2008.02.001 18374402 PMC2398769

[B27] Buckmaster P. S. Dudek F. E. (1997). Neuron loss, granule cell axon reorganization, and functional changes in the dentate gyrus of epileptic kainate-treated rats. *J. Comp. Neurol.* 385 385–404 10.1002/(SICI)1096-9861(19970901)385:3 9300766

[B28] Buckmaster P. S. Zhang G. F. Yamawaki R. (2002). Axon sprouting in a model of temporal lobe epilepsy creates a predominantly excitatory feedback circuit. *J. Neurosci.* 22 6650–6658 12151544 10.1523/JNEUROSCI.22-15-06650.2002 PMC6758164

[B29] Buterbaugh G. G. (1989). Estradiol replacement facilitates the acquisition of seizures kindled from the anterior neocortex in female rats. *Epilepsy Res.* 4 207–215 10.1016/0920-1211(89)90005-3 2612493

[B30] Carter R. B. Wood P. L. Wieland S. Hawkinson J. E. Belelli D. Lambert J. J. (1997). Characterization of the anticonvulsant properties of ganaxolone (CCD 1042; 3α-hydroxy-3β-methyl-5α-pregnan-20-one), a selective, high-affinity, steroid modulator of the γ-aminobutyric acid(A) receptor. *J. Pharmacol. Exp. Ther*. 280 1284–1295 9067315

[B31] Chisari M. Eisenman L. N. Covey D. F. Mennerick S. Zorumski C. F. (2010). The sticky issue of neurosteroids and GABAA receptors. *Trends Neurosci.* 33 299–306 10.1016/j.tins.2010.03.005 20409596 PMC2902671

[B32] Chisari M. Eisenman L. N. Krishnan K. Bandyopadhyaya A. K. Wang C. Taylor A. (2009). The influence of neuroactive steroid lipophilicity on GABAA receptor modulation: evidence for a low-affinity interaction. *J. Neurophysiol.* 102 1254–1264 10.1152/jn.00346.2009 19553485 PMC2724350

[B33] Christensen J. Kjeldsen M. J. Anderson H. Friis M. L Sidenious P. (2005). Gender differences in epilepsy. *Epilepsia* 46 956–960 10.1111/j.1528-1167.2005.51204.x 15946339

[B34] Citraro R. Russo E. Di Paola E. D. Ibbadu G. F. Gratteri S. Marra R. (2006). Effects of some neurosteroids injected into some brain areas of WAG/Rij rats, an animal model of generalized absence epilepsy. *Neuropharmacology* 50 1059–1071 10.1016/j.neuropharm.2006.02.011 16631210

[B35] Clarke R. S. Dundee J. W. Carson I. W. (1973). Proceedings: a new steroid anaesthetic-althesin. *Proc. R. Soc. Med.* 66 1027–1030 4148526 10.1177/003591577306601023 PMC1645602

[B36] Concas A. Mostallino M. C. Porcu P. Follesa P. Barbaccia M. L. Trabucchi M. (1998). Role of brain allopregnanolone in the plasticity of gamma-aminobutyric acid type A receptor in rat brain during pregnancy and after delivery. *Proc. Natl. Acad. Sci. U.S.A.* 95 13284–13289 10.1073/pnas.95.22.13284 9789080 PMC23784

[B37] Craig C. R. (1966). Anticonvulsant activity of steroids: separability of anticonvulsant from hormonal effects. *J. Pharmacol. Exp. Ther.* 153 337–343

[B38] Cutler G. B. Jr. Barnes K. M. Sauer M. A. Loriaux D. L. (1979). 11-Deoxycortisol: a glucocorticoid antagonist *in vivo*. *Endocrinology* 104 1839–1844 10.1210/endo-104-6-1839 446401

[B39] Cutler S. M. Cekic M. Miller D. M. Wali B. VanLandingham J. W. Stein D. G. (2007). Progesterone improves acute recovery after traumatic brain injury in the aged rat. *J. Neurotrauma* 24 1475–1486 10.1089/neu.2007.0294 17892409

[B40] Cutler S. M. Pettus E. H. Hoffman S. W. Stein D. G. (2005). Tapered progesterone withdrawal enhances behavioral and molecular recovery after traumatic brain injury. *Exp. Neurol.* 195 423–429 10.1016/j.expneurol.2005.06.003 16039652

[B41] de Araujo Furtado M. Rossetti F. Chanda S. Yourick D. (2012). Exposure to nerve agents: from status epilepticus to neuroinflammation, brain damage, neurogenesis and epilepsy. *Neurotoxicology* 33 1476–1490 10.1016/j.neuro.2012.09.001 23000013

[B42] DeCoster M. Lambeau G. Lazdunski M. Bazan N. G. (2002). Secreted phospholiase A2 potentiates glutamate-induced calcium increase and cell death in primary neuronal cultures. *J. Neurosci. Res.* 67 634–645 10.1002/jnr.10131 11891776

[B43] Desgent S. Duss S. Sanon N. T. Lema P. Lévesque M. Hébert D. (2012). Early-life stress is associated with gender-based vulnerability to epileptogenesis in rat pups. *PLoS ONE* 7:e42622 10.1371/journal.pone.0042622 PMC3411822 22880055

[B44] Devaud L. L. Purdy R. H. Finn D. A. Morrow A. L. (1996). Sensitization of γ-aminobutyric acidA receptors to neuroactive steroids in rats during ethanol withdrawal. *J. Pharmacol. Exp. Ther.* 278 510–517 8768698

[B45] Do Rego J. L. Seong J. Y. Burel D. Leprince J. Luu-The V. Tsutsui K. (2009). Neurosteroid biosynthesis: enzymatic pathways and neuroendocrine regulation by neurotransmitters and neuropeptides. *Front. Neuroendocrinol.* 30:259–301 10.1016/j.yfrne.2009.05.006 19505496

[B46] Dudek F. E. Staley K. J. (2011). The time course of acquired epilepsy: implications for therapeutic intervention to suppress epileptogenesis. *Neurosci. Lett.* 497 240–246 10.1016/j.neulet.2011.03.071 21458536

[B47] Dworetzky B. A. Townsend M. K. Pennell P. B. Kang J. H. (2012). Female reproductive factors and risk of seizure or epilepsy: data from the Nurses’ Health Study II. *Epilepsia* 53 1–4 10.1111/j.1528-1167.2011.03308.x PMC3253207 22050476

[B48] Edwards H. E. Burnham W. M. MacLusky N. J. (1999). Testosterone and its metabolites affect afterdischarge thresholds and the development of amygdala kindled seizures. *Brain Res.* 838 151–157 10.1016/S0006-8993(99)01620-0 10446327

[B49] Edwards H. E. Epps T. Carlen P. L. J. MacLusky N. (2000). Progestin receptors mediate progesterone suppression of epileptiform activity in tetanized hippocampal slices in vitro. *Neuroscience* 101 895–906 10.1016/S0306-4522(00)00439-5 11113338

[B50] Edwards H. E. Mo V. Burnham W. M. MacLusky N. J. (2001). Gonadectomy unmasks an inhibitory effect of progesterone on amygdala kindling in male rats. *Brain Res.* 889 260–263 10.1016/S0006-8993(00)03147-4 11166716

[B51] El-Khayat H. A. Shatla H. M. Ali G. K. Abdulgani M. O. Tomoum H. Y. Attya H. A. (2003). Physical and hormonal profile of male sexual development in epilepsy. *Epilepsia* 44 447–452 10.1046/j.1528-1157.2003.26502.x 12614402

[B52] Engel J. Pedley T. A. Aicardy J. Dichter M. A. (2007). *Epilepsy: A Comprehensive Textbook*, 2nd Edn. Philadelphia: Lippincott Williams & Wilkins

[B53] Fisher R. S. van Emde Boas W. Blume W. Elger C. Genton P. Lee P. (2005). Epileptic seizures and epilepsy: definitions proposed by the international league against epilepsy (ILAE) and the international bureau for epilepsy (IBE). *Epilepsia* 46 470–472 10.1111/j.0013-9580.2005.66104.x 15816939

[B54] Frucht M. M. Quigg M. Schwaner C. Fountain N. B. (2000). Distribution of seizure precipitants among epilepsy syndromes. *Epilepsia* 41 1534–1539 10.1111/j.1499-1654.2000.001534.x 11114210

[B55] Frye C. A. (1995). The neuroactive steroid 3 α, 5 α-THP has anti-seizure and possible neuroprotective effects in an animal model of epilepsy. *Brain Res.* 696 113–120 10.1016/0006-8993(95)00793-P 8574658

[B56] Frye C. A. Reed T. A. (1998). Androgenic neurosteroids: anti-seizure effects in an animal model of epilepsy. *Psychoneuroendocrinology* 23 385–399 10.1016/S0306-4530(98)00009-2 9695138

[B57] Frye C. A. Rhodes M. E. Walf A. A. Harney J. P. (2001a). Testosterone reduces pentylenetetrazole-induced ictal activity of wildtype mice but not those deficient in type I 5α-reductase. *Brain Res.* 918 182–186 10.1016/S0006-8993(01)02967-5 11684057

[B58] Frye C. A. Park D. Tanaka M. Rosellini R. Svare B. (2001b). The testosterone metabolite and neurosteroid 3α-androstanediol may mediate the effects of testosterone on conditioned place preference. *Psychoneuroendocrinology* 26 731–750 10.1016/S0306-4530(01)00027-0 11500254

[B59] Frye C. A. Rhodes M. E. Walf A. Harney J. (2002). Progesterone reduces pentylenetetrazol-induced ictal activity of wild-type mice but not those deficient in type I 5α -reductase. *Epilepsia* 43(Suppl. 5) 14–17 10.1046/j.1528-1157.43.s.5.19.x 12121288

[B60] Frye C. A. Scalise T. J. (2000). Anti-seizure effects of progesterone and 3α ,5α -THP in kainic acid and perforant pathway models of epilepsy. *Psychoneuroendocrinology* 25 407–420 10.1016/S0306-4530(99)00068-2 10725616

[B61] Gangisetty O. Reddy D. S. (2010). Neurosteroid withdrawal regulates GABA-A receptor α 4-subunit expression and seizure susceptibility by activation of progesterone receptor-independent early growth response factor-3 pathway. *Neuroscience* 170 865–880 10.1016/j.neuroscience.2010.07.037 20670676 PMC2939139

[B62] Gee K. W. Bolger M. B. Brinton R. E. Coirini H. McEwen B. S. (1988). Steroid modulation of the chloride ionophore in rat brain: structure-activity requirements, regional dependence and mechanism of action. *J. Pharmacol. Exp. Ther.* 246 803–812 2841455

[B63] Gibson C. L. Gray L. J. Bath P. M. Murphy S. P. (2008). Progesterone for the treatment of experimental brain injury; a systematic review. *Brain* 131 318–328 10.1093/brain/awm183 17715141

[B64] Glauser T. Ben-Menachem E. Bourgeois B. Cnaan A. Chadwick D. Guerreiro C. (2006). ILAE treatment guidelines: evidence-based analysis of antiepileptic drug efficacy and effectiveness as initial monotherapy for epileptic seizures and syndromes. *Epilepsia* 47 1094–1120 10.1111/j.1528-1167.2006.00585.x 16886973

[B65] Glauser T. Ben-Menachem E. Bourgeois B. Cnaan A. Guerreiro C. Kälviäinen R. (2013). Updated ILAE evidence review of antiepileptic drug efficacy and effectiveness as initial monotherapy for epileptic seizures and syndromes. *Epilepsia* 54 551–563 23350722 10.1111/epi.12074

[B66] Glien M. Brandt C. Potschka H. Voigt H. Ebert U. Löscher W. (2001). Repeated low-dose treatment of rats with pilocarpine: low mortality but high proportion of rats developing epilepsy. *Epilepsy Res.* 46 11–119 10.1016/s0920-1211(01)00272-8 11463512

[B67] Goddard G. V. McIntyre D. C. Leech C. K. (1969). A permanent change in brain function resulting from daily electrical stimulation. *Exp. Neurol.* 25 295–330 10.1016/0014-4886(69)90128-9 4981856

[B68] Gowers W. R. (1881). *Epilepsy and Other Chronic Convulsive Diseases*. London: Churchill

[B69] Green C. J. Halsey M. J. Precious S. Wardley-Smith B. (1978). Alphaxolone-alphadolone anesthesia in laboratory animals. *Lab. Anim.* 12 85–89 672181 10.1258/002367778780953206

[B70] Grigorian V. Z. Khudaverkian D. N. (1970). Effect of castration and subsequent administration of testosterone propionate on susceptibility to convulsions in animals. *Zh. Eksp. Klin. Med.* 10 11–17 5457174

[B71] Gyermek L. Genther G. Fleming N. (1967). Some effects of progesterone and related steroids on the central nervous system. *Int. J. Neuropharmacol.* 6 191–198 10.1016/0028-3908(67)90005-6 4382558

[B72] Harden C. MacLusky N. J. (2004). Aromatase inhibition, testosterone, and seizures. *Epilepsy Behav.* 5 260–263 10.1016/j.yebeh.2003.12.001 15123030

[B73] Harden C. MacLusky N. J. (2005). Aromatase inhibitors as add-on treatment for men with epilepsy. *Expert Rev. Neurother.* 5 123–127 10.1586/14737175.5.1.123 15853482

[B74] Harrison N. L. Majewska M. D. Harrington J. W. Barker J. L. (1987). Structure-activity relationships for steroid interactions with the γ-aminobutyric acidA receptor complex. *J. Pharmacol. Exp. Ther.* 241 346–353 3033209

[B75] Harrison N. L. Simmonds M. A. (1984). Modulation of the GABA receptor complex by a steroid anaesthetic. *Brain Res.* 323 287–292 10.1016/0006-8993(84)90299-3 6098342

[B76] Hauser W. A. (1997). “Incidence and prevalence,” in *Epilepsy, A Comprehensive Textbook* eds Engel J. Jr. Pedley T. A. (Philadelphia: Lippincott-Raven Publishers) 47–57

[B77] Hauser W. A. Annegers J. F. Kurland L. T. (1993). Incidence of epilepsy and unprovoked seizures in Rochester Minnesota during 1935–1984. *Epilepsia* 34 453–468 10.1111/j.1528-1157.1993.tb02586.x 8504780

[B78] He X.-P. Kotloski R. Nef S. Luikart B. W. Parada L. F. McNamara J. O. (2004). Conditional deletion of TrkB but not BDNF prevents epileptogenesis in the kindling model. *Neuron* 43 31–42 10.1016/j.neuron.2004.06.019 15233915

[B79] Hellier J. L. Patrylo P. R. Dou P. Nett M. Rose G. M. Dudek F. E. (1999). Assessment of inhibition and epileptiform activity in the septal dentate gyrus of freely behaving rats during the first week after kainate treatment. *J. Neurosci.* 19 10053–10064 10559413 10.1523/JNEUROSCI.19-22-10053.1999 PMC6782973

[B80] Herzog A. G. (1991). Reproductive endocrine considerations and hormonal therapy for men with epilepsy. *Epilepsia* 32(Suppl. 6) S34–S37 10.1111/j.1528-1157.1991.tb05890.x 1959510

[B81] Herzog A. G. (1995). Progesterone therapy in women with complex partial and secondary generalized seizures. *Neurology* 45 1600–1662 10.1212/WNL.45.9.1660 7675223

[B82] Herzog A. G. (1999). Progesterone therapy in women with epilepsy: a 3-year follow-up. *Neurology* 52 1917–1918 10.1212/WNL.52.9.1917-a 10371551

[B83] Herzog A. G. (2002). Altered reproductive endocrine regulation in men with epilepsy: implications for reproductive function and seizures. *Ann. Neurol.* 51 539–542 10.1002/ana.10230 12112098

[B84] Herzog A. G. Fowler K. M. Smithson S. D. Kalayjian L. A. Heck C. N. Sperling M. R. (2012). Progesterone vs placebo therapy for women with epilepsy: a randomized clinical trial. *Neurology* 78 1959–1966 10.1212/WNL.0b013e318259e1f9 22649214 PMC3369508

[B85] Herzog A. G. Frye C. A. (2003). Seizure exacerbation associated with inhibition of progesterone metabolism. *Ann. Neurol.* 53 390–391 10.1002/ana.10508 12601707

[B86] Herzog A. G. Klein P. Jacobs A. R. (1998). Testosterone versus testosterone and testolactone in treating reproductive and sexual dysfunction in men with epilepsy and hypogonadism. *Neurology* 50 782–784 10.1212/WNL.50.3.782 9521275

[B87] Herzog A. G. Klein P. Ransil B. J. (1997). Three patterns of catamenial epilepsy. *Epilepsia* 38 1082–1088 10.1111/j.1528-1157.1997.tb01197.x 9579954

[B88] Hesdorffer D. C. Beck V. Begley C. E. Bishop M. L. Cushner-Weinstein S. Holmes G. L. (2013). Research implications of the Institute of Medicine Report, Epilepsy Across the Spectrum: promoting health and understanding. *Epilepsia* 54 207–216 10.1111/epi.12056 23294462 PMC3566357

[B89] Heuser G. Eidelberg E. (1961). Steroid-induced convulsions in experimental animals. *Endocrinology* 69 915–924 10.1210/endo-69-5-915 13907062

[B90] Holmes G. L. Weber D. A. (1984). The effect of progesterone on kindling: a developmental study. *Brain Res.* 318 45–53 6488053 10.1016/0165-3806(84)90061-0

[B91] Hom A. C. Buterbaugh G. G. (1986). Estrogen alters the acquisition of seizures kindled by repeated amygdala stimulation or pentylenetetrazol administration in ovariectomized female rats. *Epilepsia* 27 103–108 10.1111/j.1528-1157.1986.tb03510.x 3956449

[B92] Hosie A. D. Wilkins M. E. da Silva H. M. A. Smart T. G. (2006). Endogenous neurosteroids regulate GABAA receptors through two discrete transmembrane sites. *Nature* 444 486–489 10.1038/nature05324 17108970

[B93] Hosie A. M. Clarke L. da Silva H. Smart T. G. (2009). Conserved site for neurosteroid modulation of GABAA receptors. *Neuropharmacology* 56 149–154 10.1016/j.neuropharm.2008.07.050 18762201

[B94] Hosie A. M. Wilkins M. E. Smart T. G. (2007). Neurosteroid binding sites on GABA-A receptors. *Pharmacol. Ther.* 116 7–19 10.1016/j.pharmthera.2007.03.011 17560657

[B95] Isojarvi J. I. Pakarinen A. J. Myllyla V. V. (1988). Effects of carbamazepine therapy on serum sex hormone levels in male patients with epilepsy. *Epilepsia* 29 781–786 10.1111/j.1528-1157.1988.tb04235.x 3191895

[B96] Jacobs M. P. Fischbach G. D. Davis M. R. Dichter M. A. Dingledine R. Lowenstein D. H. (2001). Future directions for epilepsy research. *Neurology* 57 1536–1542 10.1212/WNL.57.9.1536 11706087

[B97] Jacobs M. P. Leblanc G. G. Brooks-Kayal A. Jensen F. E. Lowenstein D. H. Noebels J. L. (2009). Curing epilepsy: progress and future directions. *Epilepsy Behav.* 14 438–445 10.1016/j.yebeh.2009.02.036 19341977 PMC2822433

[B98] Jacono J. J. Robinson J. (1987). The effects of estrogen, progesterone, and ionized calcium on seizures during the menstrual cycle in epileptic women. *Epilepsia* 28 571–577 10.1111/j.1528-1157.1987.tb03690.x 3653063

[B99] Jiang N. Chopp M. Stein D. Feit H. (1996). Progesterone is neuroprotective after transient middle cerebral artery occlusion in male rats. *Brain Res.* 735 101–107 10.1016/0006-8993(96)00605-1 8905174

[B100] Joëls M. (1997). Steroid hormones and excitability in the mammalian brain. *Front. Neuroendocrinol.* 18:2–48 10.1006/frne.1996.0144 9000458

[B101] Joëls M. (2009). Stress, the hippocampus, and epilepsy. *Epilepsia* 50 586–597 19054412 10.1111/j.1528-1167.2008.01902.x

[B102] Jones N. C. Lee H. E. Yang M. Rees S. M. Morris M. J. O’Brien T. J. (2013). Repeatedly stressed rats have enhanced vulnerability to amygdala kindling epileptogenesis. *Psychoneuroendocrinology* 38 263–270 10.1016/j.psyneuen.2012.06.005 22749310

[B103] Kaminski R. M. Fu Z. Venkatesan K. Mazzuferi M. Leclercq K. Seutin V. (2011). 11-Deoxycortisol impedes GABAergic neurotransmission and induces drug-resistant status epilepticus in mice. *Neuropharmacology* 60 1098–1108 10.1016/j.neuropharm.2010.09.021 20883706 PMC3033465

[B104] Kaminski R. M. Livingood M. R. Rogawski M. A. (2004). Allopregnanolone analogs that positively modulate GABA receptors protect against partial seizures induced by 6-Hz electrical stimulation in mice. *Epilepsia* 45 864–877 10.1111/j.0013-9580.2004.04504.x 15230714

[B105] Kaminski R. M. Marini H. Kim W. J. Rogawski M. A. (2005). Anticonvulsant activity of androsterone and etiocholanolone. *Epilepsia* 46 819–827 10.1111/j.1528-1167.2005.00705.x 15946323 PMC1181535

[B106] Karst H. de Kloet E. R Joëls M. (1999). Episodic corticosterone treatment accelerates kindling epileptogenesis and triggers long-term changes in hippocampal CA1 cells, in the fully kindled state. *Eur. J. Neurosci.* 11 889–898 10103082 10.1046/j.1460-9568.1999.00495.x

[B107] Koenig H. Schumacher M. Ferzaz B. Do Thi A. N. Ressouches A. Guennoun R. (1995). Progesterone synthesis and myelin formation by Schwann cells. *Science* 268 1500–1503 10.1126/science.7770777 7770777

[B108] Köhling R. Staley K. (2011). Network mechanisms for fast ripple activity in epileptic tissue. *Epilepsy Res.* 97 318–323 21470826 10.1016/j.eplepsyres.2011.03.006 PMC3152631

[B109] Kokate T. G. Banks M. K. Magee T. Yamaguchi S. Rogawski M. A. (1999a). Finasteride, a 5α -reductase inhibitor, blocks the anticonvulsant activity of progesterone in mice. *J. Pharmacol. Exp. Ther.* 288 679–684 9918575

[B110] Kokate T. G. Juhng K. N. Kirkby R. D. Llamas J. Yamaguchi S. Rogawski M. A. (1999b). Convulsant actions of the neurosteroid pregnenolone sulfate in mice. *Brain Res.* 831 119–124 10.1016/S0006-8993(99)01287-1 10411990

[B111] Kokate T. G. Svensson B. E. Rogawski M. A. (1994). Anticonvulsant activity of neuroactive steroids: correlation with γ-aminobutyric acid-evoked chloride current potentiation. *J. Pharmacol. Exp. Ther.* 270 1223–1229 7932175

[B112] Kokate T. G. Yamaguchi S. Pannell L. K. Rajamani U. Carroll D. M. Grossman A. B. (1998). Lack of anticonvulsant tolerance to the neuroactive steroid pregnanolone in mice. *J. Pharmacol. Exp. Ther.* 287 553–558 9808680

[B113] Kulkarni S. K. Reddy D. S. (1995). Neurosteroids: a new class of neuromodulators. *Drugs Today.* 31 433–455

[B114] Kumar G. Couper A. O’Brien T. J. Salzberg M. R. Jones N. C. Rees S. M. (2007). The acceleration of amygdala kindling epileptogenesis by chronic low-dose corticosterone involves both mineralocorticoid and glucocorticoid receptors. *Psychoneuroendocrinology* 32 834–842 10.1016/j.psyneuen.2007.05.011 17614213

[B115] Kumar G. Jones N. C. Morris M. J. Rees S. O’Brien T. J. Salzberg M. R. (2011). Early life stress enhancement of limbic epileptogenesis in adult rats: mechanistic insights. *PLoS ONE* 6:e24033 10.1371/journal.pone.0024033 PMC3177819 21957442

[B116] Lai M. C. Lui C. C. Yang S. N. Wang J. Y. Huang L. T. (2009). Epileptogenesis is increased in rats with neonatal isolation and early-life seizure and ameliorated by MK-801: a long-term MRI and histological study. *Pediatr. Res.* 66 441–447 10.1203/PDR.0b013e3181b337d2 19581840

[B117] Lambert J. J. Cooper M. A. Simmons R. D. Weir C. J. Belelli D. (2009). Neurosteroids: endogenous allosteric modulators of GABA-A receptors. *Psychoneuroendocrinology* 34(Suppl. 1) S48–58 10.1016/j.psyneuen.2009.08.009 19758761

[B118] Lawrence C. Martin B. S. Sun C. Williamson J. Kapur J. (2010). Endogenous neurosteroid synthesis modulates seizure frequency. *Ann. Neurol.* 67 689–693 20437568 10.1002/ana.21989 PMC2918659

[B119] Lévesque M. Bortel A. Gotman J. Avoli M. (2011). High-frequency (80-500 Hz) oscillations and epileptogenesis in temporal lobe epilepsy. *Neurobiol. Dis.* 42 231–241 21238589 10.1016/j.nbd.2011.01.007 PMC4873283

[B120] Logothetis J. Harner R. Morrel F. (1959). The role of estrogens in catamenial exacerbation of epilepsy. *Neurology* 9 352–360 10.1212/WNL.9.5.352 13657294

[B121] Lonsdale D. Burnham W. M. (2003). The anticonvulsant effects of progesterone and 5α-dihydroprogesterone on amygdala-kindled seizures in rats. *Epilepsia* 44 1494–1499 10.1111/j.0013-9580.2003.59402.x 14636318

[B122] Löscher W. (2002). Animal models of epilepsy for the development of antiepileptogenic and disease-modifying drugs. A comparison of the pharmacology of kindling and post-status epilepticus models of temporal lobe epilepsy. *Epilepsy Res.* 50 105–123 12151122 10.1016/s0920-1211(02)00073-6

[B123] Löscher W. (2012). “Strategies for antiepileptogenesis: antiepileptic drugs versus novel approaches evaluated in post-status epilepticus models of temporal lobe epilepsy,” in *Jasper’s Basic Mechanisms of the Epilepsies [Internet]*, 4th Edn eds Noebels J. L. Avoli M. Rogawski M. A. Olsen R. W. Delgado-Escueta A. V. (Bethesda, MD: NCBI) 22787664

[B124] MacLusky N. J. Walters M. J. Clark A. S. Toran-Allerand C. D. (1994). Aromatase in the cerebral cortex, hippocampus, and mid-brain: ontogeny and developmental implications. *Mol. Cell. Neurosci.* 5 691–698 10.1006/mcne.1994.1083 7704444

[B125] Macphee G. J. Larkin J. G. Butler E. Beastall G. H. Brodie M. J. (1988). Circulating hormones and pituitary responsiveness in young epileptic men receiving long-term antiepileptic medication. *Epilepsia* 29 468–475 10.1111/j.1528-1157.1988.tb03747.x 3134193

[B126] Majewska M. D. (1992). Neuroactive steroids: endogenous bimodal modulators of the GABA-A receptor. Mechanism of action and physiological significance. *Progr. Neurobiol.* 38 379–395 10.1016/0301-0082(92)90025-A 1349441

[B127] Majewska M. D. Demirgoren S Spivak C. E. London E. D. (1990). The neuroactive steroid dehydroepiandrosterone sulfate is an allosteric antagonist of the GABA-A receptor. *Brain Res.* 526 143–146 10.1016/0006-8993(90)90261-9 1964106

[B128] Majewska M. D. Harrison N. L. Schwartz R. D. Barker J. L. Paul S. M. (1986). Steroid hormone metabolites are barbiturate-like modulators of the GABA receptor. *Science* 232 1004–1007 10.1126/science.2422758 2422758

[B129] Majewska M. D. Schwartz R. D. (1987). Pregnenolone sulfate: an endogenous antagonist of the γ-aminobutyric acid receptor complex in brain? *Brain Res.* 404 355–360 10.1016/0006-8993(87)91394-1 3032339

[B130] Mani R. Pollard J. Dichter M. A. (2011). Human clinical trails in antiepileptogenesis. *Neurosci. Lett.* 497 251–256 10.1016/j.neulet.2011.03.010 21439351 PMC3138332

[B131] McClelland S. Dubé C. M. Yang J. Baram T. Z. (2011). Epileptogenesis after prolonged febrile seizures: mechanisms, biomarkers and therapeutic opportunities. *Neurosci. Lett.* 497 155–162 21356275 10.1016/j.neulet.2011.02.032 PMC3109205

[B132] McHugh J. C. Delanty N. (2008). Epidemiology and classification of epilepsy, gender comparisons. *Int. Rev. Neurobiol.* 83 11–26 10.1016/S0074-7742(08)00002-0 18929074

[B133] McNamara J. O. Morrisett R. Nadler J. V. (1992). Recent advances in understanding mechanisms of the kindling model. *Adv. Neurol.* 57 555–560 1543080

[B134] Meffre D. Pianos A. Liere P. Eychenne B. Cambourg A. Schumacher M. (2007). Steroid profiling in brain and plasma of male and pseudopregnant female rats after traumatic brain injury: analysis by gas chromatography/mass spectrometry. *Endocrinology* 148 2505–2517 17303653 10.1210/en.2006-1678

[B135] Meyer R. P. Hagemeyer C. E. Knoth R. Kaufmann M. R. Volk B. (2006). Anti-epileptic drug phenytoin enhances androgen metabolism and androgen receptor expression in murine hippocampus. *J. Neurochem.* 96 460–472 10.1111/j.1471-4159.2005.03555.x 16336225

[B136] Mienville J. M. Vicini S. (1989). Pregnenolone sulfate antagonizes GABA-A receptor-mediated currents via a reduction of channel opening frequency. *Brain Res.* 489 190–194 10.1016/0006-8993(89)90024-3 2472854

[B137] Mihalek R. M. Banerjee P. K. Korpi E. R. Quinlan J. J. Firestone L. L Mi Z. P. (1999). Attenuated sensitivity to neuroactive steroids in γ-aminobutyrate type A receptor δ subunit knockout mice. *Proc. Natl. Acad. Sci. U.S.A.* 96 12905–12910 10.1073/pnas.96.22.12905 10536021 PMC23157

[B138] Mohammad S. Abolhassan A. Pourgholami M. H. (1998). Evaluation of the anticonvulsant profile of progesterone in male amygdala-kindled rats. *Epilepsy Res.* 30 195–202 10.1016/S0920-1211(98)00004-7 9657647

[B139] Morimoto K. Fahnestock M. Racine R. J. (2004). Kindling and status epilepticus models of epilepsy: rewiring the brain. *Prog. Neurobiol*. 73 1–60 10.1016/j.pneurobio.2004.03.009 15193778

[B140] Mtchedlishvili Z. Bertram E. H. Kapur J. (2001). Diminished allopregnanolone enhancement of GABA-A receptor currents in a rat model of chronic temporal lobe epilepsy. *J. Physiol.* 537(Pt 2) 453–465 10.1111/j.1469-7793.2001.00453.x 11731578 PMC2278949

[B141] Mtchedlishvili Z. Kapur J. (2003). A presynaptic action of the neurosteroid pregnenolone sulfate on GABAergic synaptic transmission. *Mol. Pharmacol.* 64 857–864 10.1124/mol.64.4.857 14500742

[B142] Murashima Y. L. Yoshii M. (2010). New therapeutic approaches for epilepsies, focusing on reorganization of the GABA-A receptor subunits by neurosteroids. *Epilepsia* 51(Supp l3) 131–134 10.1111/j.1528-1167.2010.02627.x 20618418

[B143] Nadler J. V. (2003). The recurrent mossy fiber pathway of the epileptic brain. *Neurochem. Res.* 28 1649–1658 10.1023/A:1026004904199 14584819

[B144] Norwood B. A. Bumanglag A. V. Osculati F. Sbarbati A. Marzola P. Nicolato E. (2010). Classic hippocampal sclerosis and hippocampal-onset epilepsy produced by a single “cryptic” episode of focal hippocampal excitation in awake rats. *J. Comp. Neurol.* 518 3381–3407 10.1002/cne.22406 20575073 PMC2894278

[B145] Nothdurfter C. Rammes G. Baghai T. C. Schüle C. Schumacher M. Papadopoulos V. (2011). TSPO (18 kDa) as a target for novel anxiolytics with a favourable side-effect profile. *J. Neuroendocrinol.* 24 82–92 21609361 10.1111/j.1365-2826.2011.02166.x

[B146] O’Dell C. M. Das A. Wallace G. IV Ray S. K. Banik N. L. (2012). Understanding the basic mechanisms underlying seizures in mesial temporal lobe epilepsy and possible therapeutic targets, a review. *J. Neurosci. Res.* 90 913–924 10.1002/jnr.22829 22315182 PMC11877321

[B147] Park-Chung M. Malayev A. Purdy R. H. Gibbs T. T. Farb D. H. (1999). Sulfated and unsulfated steroids modulate γ-aminobutyric acidA receptor function through distinct sites. *Brain Res.* 830 72–87 10.1016/S0006-8993(99)01381-5 10350561

[B148] Patel M. (2004). Mitochondrial dysfunction and oxidative stress, cause and consequences of epileptic seizures. *Free Radic. Biol. Med.* 37 1951–1962 10.1016/j.freeradbiomed.2004.08.021 15544915

[B149] Peng Z. Huang C. S. Stell B. M. Mody I. Houser C. R. (2004). Altered expression of the δ subunit of the GABAA receptor in a mouse model of temporal lobe epilepsy. *J. Neurosci.* 24 8629–8639 10.1523/JNEUROSCI.2877-04.2004 15456836 PMC6729896

[B150] Pericić D. Svob D. Jazvinsćak M. Mirković K. (2000). Anticonvulsive effect of swim stress in mice. *Pharmacol. Biochem. Behav.* 66 879–886 10.1016/S0091-3057(00)00267-7 10973529

[B151] Pericic D. Manev H. Bujas M. (1996). Gonadal hormones and picrotoxin-induced convulsions in male and female rats. *Brain Res.* 736 174–179 10.1016/0006-8993(96)00677-4 8930322

[B152] Pesce M. E. Acevedo X. Bustamante D. Miranda H. E. Pinardi G. (2000). Progesterone and testosterone modulate the convulsant actions of pentylenetetrazol and strychnine in mice. *Pharmacol. Toxicol.* 87 116–119 10.1111/j.0901-9928.2000.870303.x 11068851

[B153] Pitkänen A. Immonen R. J. Gröhn O. H. Kharatishvili I. (2009). From traumatic brain injury to posttraumatic epilepsy, what animal models tell us about the process and treatment options. *Epilepsia * 50(Suppl.2) 1–29 10.1111/j.1528-1167.2008.02007.x 19187291

[B154] Pitkänen A. Lukasiuk K. (2011). Mechanisms of epileptogenesis and potential treatment targets. *Lancet Neurol.* 10 173–186 21256455 10.1016/S1474-4422(10)70310-0

[B155] Pugnaghi M. Monti G. Biagini G. Meletti S. (2013). Temporal lobe epilepsy exacerbation during pharmacological inhibition of endogenous neurosteroid synthesis. *BMJ Case Rep. 2013.* pii: bBR2012008204 10.1136/bcr-2012-008204 PMC3604425 23425566

[B156] Purdy R. H. Morrow A. L. Blinn J. R. Paul S. M. (1990). Synthesis, metabolism, and pharmacological activity of 3α-hydroxy steroids which potentiate GABA-receptor-mediated chloride ion uptake in rat cerebral cortical synaptoneurosomes. *J. Med. Chem.* 33 1572–1581 10.1021/jm00168a008 2160534

[B157] Purdy R. H. Morrow A. L. Moore P. H. Jr. Paul S. M. (1991). Stress-induced elevations of γ-aminobutyric acid type A receptor-active steroids in the rat brain. *Proc. Natl. Acad. Sci. U.S.A.* 88 4553–4557 10.1073/pnas.88.10.4553 1852011 PMC51699

[B158] Ramakrishnan L. Hess G. P. (2010). Mechanism of potentiation of a dysfunctional epilepsy-linked mutated GABA(A) receptor by a neurosteroid (3α,21-dihydroxy-5α-pregnan-20-one), transient kinetic investigations. *Biochemistry* 49 7892–7901 10.1021/bi901241g 20726514

[B159] Ramanathan G. Reddy D. S. (2011). Inhibition of endogenous neurosteroids accelerates limbic epileptogenesis. *Epilepsy Curr.* 2(Suppl. 1) Abst. 1.022

[B160] Rao M. S. Hattiangady B. Reddy D. S. Shetty A. K. (2006). Hippocampal neurodegeneration, spontaneous seizures, and mossy fiber sprouting in the F344 rat model of temporal lobe epilepsy. *J. Neurosci. Res.* 83 1088–1105 10.1002/jnr.20802 16493685

[B161] Reddy D. S. (2003a). Pharmacology of endogenous neuroactive steroids. *Crit. Rev. Neurobiology* 15 197–234 10.1615/CritRevNeurobiol.v15.i34.20 15248811

[B162] Reddy D. S. (2003b). Is there a physiological role for the neurosteroid THDOC in stress-sensitive conditions? *Trends Pharmacol. Sci.* 24 103–106 10.1016/S0165-6147(03)00023-3 12628349

[B163] Reddy D. S. (2004a). Anticonvulsant activity of the testosterone-derived neurosteroid 3alpha-androstanediol. *Neuroreport* 15 515–518 10.1097/00001756-200403010-00026 15094514

[B164] Reddy D. S. (2004b). Testosterone modulation of seizure susceptibility is mediated by neurosteroids 3α-androstanediol and 17β-estradiol. *Neuroscience* 129 195–207 10.1016/j.neuroscience.2004.08.002 15489042

[B165] Reddy D. S. (2006). Physiological role of adrenal deoxycorticosterone-derived neuroactive steroids in stress-sensitive conditions. *Neuroscience* 138 911–920 10.1016/j.neuroscience.2005.10.016 16325348

[B166] Reddy D. S. (2008). Mass spectrometric quantification and physiological-pharmacological activity of androgenic neurosteroids. *Neurochem. Int.* 52 541–553 10.1016/j.neuint.2007.05.019 17624627 PMC2390862

[B167] Reddy D. S. (2009a). The role of neurosteroids in the pathophysiology and treatment of catamenial epilepsy. *Epilepsy Res.* 85 1–30 10.1016/j.eplepsyres.2009.02.017 19406620 PMC2696558

[B168] Reddy D. S. (2009b). “Steroid hormones and sex differences in seizure susceptibility,” in *Encyclopedia of Basic Epilepsy Research* Vol. 1 ed. Schwartzkroin Philip (Oxford: Academic Press) 526–533 10.1016/B978-012373961-2.00157-0

[B169] Reddy D. S. (2010). Neurosteroids, Endogenous role in the human brain and therapeutic potentials. *Prog. Brain Res.* 186 113–137 10.1016/B978-0-444-53630-3.00008-7 21094889 PMC3139029

[B170] Reddy D. S. (2011). Role of anticonvulsant and antiepileptogenic neurosteroids in the pathophysiology and treatment of epilepsy. *Front. Endocrinol.* 2:38 10.3389/fendo.2011.00038 PMC3356070 22654805

[B171] Reddy D. S. (2013). Neuroendocrine aspects of catamenial epilepsy. *Horm. Behav.* 63 254–266 10.1016/j.yhbeh.2012.04.016 22579656 PMC3422425

[B172] Reddy D. S. Castaneda D. C. O’Malley B. W. Rogawski M. A. (2004). Anticonvulsant activity of progesterone and neurosteroids in progesterone receptor knockout mice. *J. Pharmacol. Exp. Ther.* 310 230–239 10.1124/jpet.104.065268 14982969

[B173] Reddy D. S. Gangisetty O. Briyal S. (2010). Disease-modifying activity of progesterone in the hippocampus kindling model of epileptogenesis. *Neuropharmacology* 59 573–581 10.1016/j.neuropharm.2010.08.017 20804775 PMC2963708

[B174] Reddy D. S. Gould J. Gangisetty O. (2012). A mouse kindling model of perimenstrual catamenial epilepsy. *J. Pharmacol. Exp. Ther.* 341 784–793 10.1124/jpet.112.192377 22434675 PMC3362885

[B175] Reddy D. S. Jian K. (2010). The testosterone-derived neurosteroid androstanediol is a positive allosteric modulator of GABA-A receptors. *J. Pharmacol. Exp. Ther.* 334 1031–1041 10.1124/jpet.110.169854 20551294 PMC2939675

[B176] Reddy D. S. Kim H. Y. Rogawski M. A. (2001). Neurosteroid withdrawal model of perimenstrual catamenial epilepsy. *Epilepsia* 42 328–336 10.1046/j.1528-1157.2001.10100.x 11442149

[B177] Reddy D. S. Kulkarni S. K. (1998). Proconvulsant effects of neurosteroids pregnenolone sulfate and dehydroepiandrosterone sulfate in mice. *Eur. J. Pharmacol.* 345 55–59 10.1016/S0014-2999(98)0034-X 9593594

[B178] Reddy D. S. Kulkarni S. K. (2000). Development of neuroactive steroid-based novel psychotropic drugs. *Prog. Med. Chem.* 37 135–175 10.1016/S0079-6468(08)70059-6 10845249

[B179] Reddy D. S. Mohan A. (2011). Development and persistence of limbic epileptogenesis are impaired in mice lacking progesterone receptors. *J. Neurosci.* 31 650–658 10.1523/JNEUROSCI.4488-10.2011 21228174 PMC6623446

[B180] Reddy D. S. O’Malley B. W. Rogawski M. A. (2005). Anxiolytic activity of progesterone in progesterone receptor knockout mice. *Neuropharmacology* 48 14–24 10.1016/j.neuropharm.2004.09.002 15617723

[B181] Reddy D. S. Ramanathan G. (2012). Finasteride inhibits the disease-modifying activity of progesterone in the hippocampus kindling model of epileptogenesis. *Epilepsy Behav.* 25 92–97 10.1016/j.yebeh.2012.05.024 22835430 PMC3444667

[B182] Reddy D. S. Rogawski M. A. (2000). Chronic treatment with the neuroactive steroid ganaxolone in the rat induces anticonvulsant tolerance to diazepam but not to itself. *J. Pharmacol. Exp. Ther.* 295 1241–1248 11082461

[B183] Reddy D. S. Rogawski M. A. (2001). Enhanced anticonvulsant activity of neuroactive steroids in a rat model of catamenial epilepsy. *Epilepsia* 42 303–310 10.1046/j.1528-1157.2001.10200.x 11442150

[B184] Reddy D. S. Rogawski M. A. (2002). Stress-induced deoxycorticosterone-derived neuroactive steroids modulates GABA(A) receptor function and seizure susceptibility. *J. Neurosci.* 42 3795–3805 11978855 10.1523/JNEUROSCI.22-09-03795.2002 PMC6758375

[B185] Reddy D. S. Rogawski M. A. (2009). Neurosteroid replacement therapy for catamenial epilepsy. *Neurotherapeutics.* 6 392–401 10.1016/j.nurt.2009.01.006 19332335 PMC2682439

[B186] Reddy D. S. Rogawski M. A. (2010). Ganaxolone suppression of behavioral and electrographic seizures in the mouse amygdala kindling model. *Epilepsy Res.* 89 254–260 10.1016/j.eplepsyres.2010.01.009 20172694 PMC2854307

[B187] Reddy D. S. Rogawski M. A. (2012). “Neurosteroids – endogenous regulators of seizure susceptibility and role in the treatment of epilepsy (Chapter 77),” in *Jasper’s Basic Mechanisms of the Epilepsies*, 4th Edn eds Noebels J. L. Avoli M. Rogawski M. A. Olsen R. W. Delgado-Escueta A. V. (New York, NY: Oxford University Press) 982–1000

[B188] Reddy D. S. Woodward R. (2004). Ganaxolone: a prospective overview. *Drugs Future* 29 227–242 10.1358/dof.2004.029.03.793135

[B189] Reibel S. ré V. Chassagnon S. ré G. Marescaux C. Nehlig A. (2000). Neuroprotective effects of chronic estradiol benzoate treatment on hippocampal cell loss induced by status epilepticus in the female rat. *Neurosci. Lett.* 281 79–82 10704747 10.1016/s0304-3940(00)00784-9

[B190] Roberts A. J. Keith L. D. (1995). Corticosteroids enhance convulsion susceptibility via central mineralocorticoid receptors. *Psychoneuroendocrinology* 20 891–902 10.1016/0306-4530(95)00016-X 8834095

[B191] Robertson C. R. Flynn S. P. White H. S. Bulaj G. (2011). Anticonvulsant neuropeptides as drug leads for neurological diseases. *Nat. Prod. Rep.* 28 741–762 10.1039/c0np00048e 21340067

[B192] Roof R. L. Duvdevani R. Braswell L. Stein D. G. (1994). Progesterone facilitates cognitive recovery and reduces secondary neuronal loss caused by cortical contusion injury in male rats. *Exp. Neurol.* 129 64–69 10.1006/exnr.1994.1147 7925843

[B193] Roof R. L. Hall E. D. (2000). Gender differences in acute CNS trauma and stroke, neuroprotective effects of estrogen and progesterone. *J. Neurotrauma.* 17 367–388 10.1089/neu.2000.17.367 10833057

[B194] Rose R. P. Morell F. Hoeppner T. J. (1979). Influences of pituitary–adrenal hormones on kindling. *Brain Res.* 169 303–315 10.1016/0006-8993(79)91032-1 445160

[B195] Rupprecht R. Papadopoulos V. Rammes G. Baghai T. C. Fan J. Akula N. (2010). Translocator protein (18 kDa) (TSPO) as a therapeutic target for neurological and psychiatric disorders. *Nat. Rev. Drug Discov.* 9 971–988 10.1038/nrd3295 21119734

[B196] Rupprecht R. Rammes G. Eser D. Baghai T. C. Schüle C. Nothdurfter C. (2009). Translocator protein (18 kD) as target for anxiolytics without benzodiazepine-like side effects. *Science* 325 490–493 19541954 10.1126/science.1175055

[B197] Rupprecht R. Reul J. M. Trapp T. van Steensel B. Wetzel C. Damm K. (1993). Progesterone receptor-mediated effects of neuroactive steroids. *Neuron* 11 523–530 10.1016/0896-6273(93)90156-L 8398145

[B198] Saalmann Y. B. Kirkcaldie M. T. Waldron S. Calford M. B. (2007). Cellular distribution of the GABA-A receptor-modulating 3α -hydroxy, 5α -reduced pregnane steroids in the adult rat brain. *J. Neuroendocrinol.* 19 272–284 10.1111/j.1365-2826.2006.01527.x 17355317

[B199] Salzberg M. Kumar G. Supit L. Jones N. C. Morris M. J. Rees S O’Brien T. J. (2007). Early postnatal stress confers enduring vulnerability to limbic epileptogenesis. *Epilepsia* 48 2079–2085 10.1111/j.1528-1167.2007.01246.x 17999745

[B200] Scharfman H. E. Kim M. Hintz T. M. MacLusky N. J. (2008). Seizures and reproductive function, insights from female rats with epilepsy. *Ann. Neurol.* 64 687–697 10.1002/ana.21518 19107990 PMC2677522

[B201] Scharfman H. E. MacLusky N. J. (2006). The influence of gonadal hormones on neuronal excitability, seizures, and epilepsy in the female. *Epilepsia* 47 1423–1440 10.1111/j.1528-1167.2006.00672.x 16981857 PMC1924802

[B202] Scharfman H. E. Malthankar-Phatak G. H. Friedman D. Pearce P. McCloskey D. P. Harden C. L. (2009). A rat model of epilepsy in women: a tool to study physiological interactions between endocrine systems and seizures. *Endocrinology* 150 4437–4442 10.1210/en.2009-0135 19443573 PMC2736077

[B203] Schwartz-Giblin S. Korotzer A. Pfaff D. W. (1989). Steroid hormone effects on picrotoxin-induced seizures in female and male rats. *Brain Res.* 476 240–247 10.1016/0006-8993(89)91244-4 2702466

[B204] Selye H. (1941). Anesthetics of steroid hormones. *Proc. Soc. Exp. Biol. Med.* 46 116–121 10.3181/00379727-46-11907

[B205] Selye H. (1942). The antagonism between anesthetic steroid hormones and pentamethylenetetrazol (metrazol). *J. Lab. Clin. Med*. 27 1051–1053

[B206] Shen H. Sabaliauskas N. Sherpa A. Fenton A. A. Stelzer A. Aoki C. (2010). A critical role for α4βδ GABA-A receptors in shaping learning deficits at puberty in mice. *Science* 327 1515–1518 10.1126/science.1184245 20299596 PMC2887350

[B207] Silva de Lacerda A. F. Janjoppi L. Scorza F. A. Lima E. Amado D. Cavalheiro E. A. (2007). Physical exercise program reverts the effects of pinealectomy on the amygdala kindling development. *Brain Res. Bull.* 74 16–220 10.1016/j.brainresbull.2007.06.011 17720542

[B208] Simonato M. Löscher W. Cole A. J. Dudek F. E. Engel J. Jr. Kaminski R. M. (2012). Finding a better drug for epilepsy: preclinical screening strategies and experimental trial design. *Epilepsia* 53 1860–1867 22708847 10.1111/j.1528-1167.2012.03541.x PMC4208688

[B209] Singh M. Su C. (2013). Progesterone and neuroprotection. *Horm. Behav.* 63 284–290 10.1016/j.yhbeh.2012.06.003 22732134 PMC3467329

[B210] Sloviter R. S. Bumanglag A. V. (2013). Defining “epileptogenesis” and identifying “antiepileptogenic targets” in animal models of acquired temporal lobe epilepsy is not as simple as it might seem. *Neuropharmacology* 69 3–15 10.1016/j.neuropharm.2012.01.022 22342985 PMC3398197

[B211] Snead O. C. III (1998). Ganaxolone, a selective, high-affinity steroid modulator of the γ-aminobutyric acid-A receptor, exacerbates seizures in animal models of absence. *Ann. Neurol.* 44 688–691 10.1002/ana.410440417 9778270

[B212] Spigelman I. Li Z. Banerjee P. K. Mihalek R. M. Homanics G. E. Olsen R. W. (2002). Behavior and physiology of mice lacking the GABA-A receptor δ subunit. *Epilepsia.* 43(Suppl. 5) 3–8 10.1046/j.1528-1157.43.s.5.8.x 12121286

[B213] Stables J. P. Bertram E. H. White H. S. Coulter D. A. Dichter M. A. Jacobs M. P. (2002). Models for epilepsy and epileptogenesis: report from the NIH workshop, Bethesda, Maryland. *Epilepsia* 43 1410–1420 10.1046/j.1528-1157.2002.06702.x 12423393

[B214] Steffensen S. C. (1995). Dehydroepiandrosterone sulfate suppresses hippocampal recurrent inhibition and synchronizes neuronal activity to theta rhythm. *Hippocampus* 5 320–328 10.1002/hipo.450050405 8589795

[B215] Stein D. G. (2013). A clinical/translational perspective: can a developmental hormone play a role in the treatment of traumatic brain injury? *Horm. Behav.* 63 291–300 10.1016/j.yhbeh.2012.05.004 22626570

[B216] Stein D. G. Sayeed I. (2010). Is progesterone worth consideration as a treatment for brain injury. *Am. J. Roentgenol.* 194 20–22 10.2214/AJR.09.3407 20028900

[B217] Stell B. M. Brickley S. G. Tang C. Y. Farrant M. Mody I. (2003). Neuroactive steroids reduce neuronal excitability by selectively enhancing tonic inhibition mediated by δ subunit-containing GABA-A receptors. *Proc. Natl. Acad. Sci. U.S.A.* 100 14439–14444 10.1073/pnas.2435457100 14623958 PMC283610

[B218] Stoffel-Wagner B. Beyenburg S. Watzka M. S. Blumcke I. Bauer J. Schramm J. (2000). Expression of 5α-reductase and 3α-hydroxysteroid oxidoreductase in the hippocampus of patients with chronic temporal lobe epilepsy. *Epilepsia* 41 140–147 10.1111/j.1528-1157.2000.tb00133.x 10691110

[B219] Stoffel-Wagner B. Watzka M. Steckelbroeck S. Ludwig M. Clusmann H. Bidlingmaier F. (2003). Allopregnanolone serum levels and expression of 5α-reductase and 3α-hydroxysteroid dehydrogenase isoforms in hippocampal and temporal cortex of patients with epilepsy. *Epilepsy Res.* 54 11–19 10.1016/S0920-1211(03)00036-6 12742591

[B220] Sun C. Mtchedlishvili Z. Erisir A. Kapur J. (2007). Diminished neurosteroid sensitivity of synaptic inhibition and altered location of the α4-subunit of GABA-A receptors in an animal model of epilepsy. *J. Neurosci.* 27 12641–12650 10.1523/JNEUROSCI.4141-07.2007 18003843 PMC2878477

[B221] Sutula T. Cascino G. Cavazos J. Parada I. Ramirez L. (1989). Mossy fiber synaptic reorganization in the epileptic human temporal lobe. *Ann. Neurol.* 26 321–330 10.1002/ana.410260303 2508534

[B222] Temkin N. R. (2001). Antiepileptogenesis and seizure prevention trials with antiepileptic drugs, meta-analysis of controlled trials. *Epilepsia* 42 515–524 10.1046/j.1528-1157.2001.28900.x 11440347

[B223] Temkin N. R. Davis G. R. (1984). Stress as a risk factor for seizures among adults with epilepsy. *Epilepsia* 25 450–456 10.1111/j.1528-1157.1984.tb03442.x 6745217

[B224] Thomas J. McLean J. H. (1991). Castration alters susceptibility of male rats to specific seizures. *Physiol. Behav.* 49 1177–1179 10.1016/0031-9384(91)90347-Q 1896499

[B225] Thomas J. Yang Y. C. (1991). Allylglycine-induced seizures in male and female rats. *Physiol. Behav.* 49 1181–1183 10.1016/0031-9384(91)90348-R 1654571

[B226] Tsuda M. Suzuki T. Misawa M. (1997). Modulation of the decrease in the seizure threshold of pentylenetetrazole in diazepam-withdrawn mice by the neuroactive steroid 5α-pregnan-3α,21-diol-20-one (alloTHDOC). *Addict. Biol.* 2 455–460 10.1080/13556219772516 26735951

[B227] Twyman R. E. Macdonald R. L. (1992). Neuroactive steroid regulation of GABA-A receptor single-channel kinetic properties of mouse spinal cord neurons in culture. *J. Physiol.* 456 215–245 1338096 10.1113/jphysiol.1992.sp019334 PMC1175679

[B228] Vallée M. Rivera J. D. Koob G. F. Purdy R. H. Fitzgerald R. L. (2000). Quantification of neuroactive steroids in rat plasma and brain following swim stress and allopregnanolone administration using negative chemical ionization gas chromatography/mass spectrometry. *Anal. Biochem.* 287 153–166 11078595 10.1006/abio.2000.4841

[B229] van Vliet E. A. Aronica E. Tolner E. A. Lopes da Silva F. H. Gorter J. A. (2004). Progression of temporal lobe epilepsy in the rat is associated with immunocytochemical changes in inhibitory interneurons in specific regions of the hippocampal formation. *Exp. Neurol.* 187 367–379 10.1016/j.expneurol.2004.01.016 15144863

[B230] Velíšková J. (2006). The role of estrogens in seizures and epilepsy: the bad guys or the good guys? *Neuroscience* 138 837–844 16310960 10.1016/j.neuroscience.2005.07.005

[B231] Velíšková J. (2007). Estrogens and Epilepsy: why are we so excited? *Neuroscientist* 13 77–88 17229977 10.1177/1073858406295827

[B232] Velíšková J. (2009). “Sex differences in seizure susceptibility,” in *Encyclopedia of Basic Epilepsy Research* ed. Schwartzkroin Philip (Oxford: Academic Press) 1–4

[B233] Velíšková J. Velísek L. (2007). Beta-estradiol increases dentate gyrus inhibition in female rats via augmentation of hilar neuropeptide-Y. *J. Neurosci.* 27 6054–6063 17537977 10.1523/JNEUROSCI.0366-07.2007 PMC6672257

[B234] Velíšková J. Velisek L. Galanopoulou A. S. Sperber E. F. (2000). Neuroprotective effects of estrogens on hippocampal cells in adult female rats after status epilepticus. *Epilepsia* 41(Suppl. 6) S30–S35 10999516 10.1111/j.1528-1157.2000.tb01553.x

[B235] Verrotti A. Latini G. Manco R. De Simone M. Chiarelli F. (2007). Influence of sex hormones on brain excitability and epilepsy. *J. Endocrinol. Invest.* 30 97–803 17993775 10.1007/BF03350821

[B236] Vezzani A. (2005). Inflammation and epilepsy. *Epilepsy Curr.* 5 1–6 10.1111/j.1535-7597.2005.05101.x 16059445 PMC1176317

[B237] Vezzani A. French J. Bartfai T. Baram T. Z. (2011). The role of inflammation in epilepsy. *Nat. Rev. Neurol.* 7 31–40 10.1038/nrneurol.2010.178 21135885 PMC3378051

[B238] VMDB Report (2003). Search terms “epilepsy” in intact versus castrated dogs. *The Veterinary Medical Database.* Available at:

[B239] Weiss G. K. Castillo N. Fernandez M. (1993). Amygdala kindling rate is altered in rats with a deficit in the responsiveness of the hypothalamo-pituitary-adrenal axis. *Neurosci. Lett.* 157 91–94 10.1016/0304-3940(93)90650-A 8233039

[B240] Wieland S. Belluzzi J. D. Stein L. Lan N. C. (1995). Comparative behavioral characterization of the neuroactive steroids 3α-OH,5α-pregnan-20-one and 3α-OH,5β-pregnan-20-one in rodents. *Psychopharmacology.* 118 65–71 10.1007/BF02245251 7597124

[B241] Wieser H. G. ILAE Commission on Neurosurgery of Epilepsy (2004). ILAE Commission Report. Mesial temporal lobe epilepsy with hippocampal sclerosis. *Epilepsia* 45 695–714 10.1111/j.0013-9580.2004.09004.x 15144438

[B242] Williams P. A. White A. M. Clark S. Ferraro D. J. Swiercz W. Staley K. J. (2009). Development of spontaneous recurrent seizures after kainate-induced status epilepticus. *J. Neurosci.* 29 2103–2112 10.1523/JNEUROSCI.0980-08.2009 19228963 PMC2897752

[B243] Williamson J. Mtchedlishvili Z. Kapur J. (2004). Characterization of the convulsant action of pregnenolone sulfate. *Neuropharmacology* 46 56–864 10.1016/j.neuropharm.2003.11.029 PMC2885607 15033345

[B244] Wohlfarth K. M. Bianchi M. T. Macdonald R. L. (2002). Enhanced neurosteroid potentiation of ternary GABAA receptors containing the δ subunit. *J. Neurosci.* 22 1541–1549 11880484 10.1523/JNEUROSCI.22-05-01541.2002 PMC6758857

[B245] Woolley C. S. (2000). Estradiol facilitates kainic acid-induced, but not flurothyl-induced, behavioral seizure activity in adult female rats. *Epilepsia* 41 510–515 10.1111/j.1528-1157.2000.tb00203.x 10802755

[B246] Wright D. W. Kellermann A. L. Hertzberg V. S. Clark P. L. Frankel M. Goldstein F. C. (2007). ProTECT: a randomized clinical trial of progesterone for acute traumatic brain injury. *Ann. Emerg. Med.* 49 391–402 10.1016/j.annemergmed.2006.07.932 17011666

[B247] Wu F. S. Gibbs T. T. Farb D. H. (1991). Pregnenolone sulfate: a positive allosteric modulator at the N-methyl-D-aspartate receptor. *Mol. Pharmacol.* 40 333–336 1654510

[B248] Xiao G. Wei J. Yan W. Wang W. Lu Z. (2008). Improved outcomes from the administration of progesterone for patients with acute severe traumatic brain injury: a randomized controlled trial. *Crit. Care* 12 R61 10.1186/cc6887 PMC2447617 18447940

[B249] Yagami T. Ueda K. Asakura K. Hayasaki-Kajiwara Y. Nakazato H. Sakaeda T. (2002). Group IB secretory phospholipase A2 induces neuronal cell death via apoptosis. *J. Neurochem.* 81 449–461 10.1046/j.1471-4159.2002.00800.x 12065654

[B250] Zhang N. Wei W. Mody I. Houser C. R. (2007). Altered localization of GABAA receptor subunits on dentate granule cell dendrites influences tonic and phasic inhibition in a mouse model of epilepsy. *J. Neurosci*. 27 7520–7531 10.1523/JNEUROSCI.1555-07.2007 17626213 PMC6672608

